#  Overexpression of 14-3-3ζ Promotes Tau Phosphorylation at Ser^262^ and Accelerates Proteosomal Degradation of Synaptophysin in Rat Primary Hippocampal Neurons

**DOI:** 10.1371/journal.pone.0084615

**Published:** 2013-12-19

**Authors:** Hamid Y. Qureshi, Dong Han, Ryen MacDonald, Hemant K. Paudel

**Affiliations:** 1 The Bloomfield Center for Research in Aging, Lady Davis Institute for Medical Research, Jewish General Hospital, Montreal, Quebec, Canada; 2 Department of Neurology and Neurosurgery, McGill University, Montreal, Quebec, Canada; Alexander Fleming Biomedical Sciences Research Center, Greece

## Abstract

β-amyloid peptide accumulation, tau hyperphosphorylation, and synapse loss are characteristic neuropathological symptoms of Alzheimer’s disease (AD). Tau hyperphosphorylation is suggested to inhibit the association of tau with microtubules, making microtubules unstable and causing neurodegeneration. The mechanism of tau phosphorylation in AD brain, therefore, is of considerable significance. Although PHF-tau is phosphorylated at over 40 Ser/Thr sites, Ser^262^ phosphorylation was shown to mediate β-amyloid neurotoxicity and formation of toxic tau lesions in the brain. *In vitro*, PKA is one of the kinases that phosphorylates tau at Ser^262^, but the mechanism by which it phosphorylates tau in AD brain is not very clear. 14-3-3ζ is associated with neurofibrillary tangles and is upregulated in AD brain. In this study, we show that 14-3-3ζ promotes tau phosphorylation at Ser^262^ by PKA in differentiating neurons. When overexpressed in rat hippocampal primary neurons, 14-3-3ζ causes an increase in Ser^262^ phosphorylation, a decrease in the amount of microtubule-bound tau, a reduction in the amount of polymerized microtubules, as well as microtubule instability. More importantly, the level of pre-synaptic protein synaptophysin was significantly reduced. Downregulation of synaptophysin in 14-3-3ζ overexpressing neurons was mitigated by inhibiting the proteosome, indicating that 14-3-3ζ promotes proteosomal degradation of synaptophysin. When 14-3-3ζ overexpressing neurons were treated with the microtubule stabilizing drug taxol, tau Ser^262^ phosphorylation decreased and synaptophysin level was restored. Our data demonstrate that overexpression of 14-3-3ζ accelerates proteosomal turnover of synaptophysin by promoting the destabilization of microtubules. Synaptophysin is involved in synapse formation and neurotransmitter release. Our results suggest that 14-3-3ζ may cause synaptic pathology by reducing synaptophysin levels in the brains of patients suffering from AD.

## Introduction

Senile plaques and neurofibrillary tangles (NFTs) are the characteristic neuropathological hallmarks found in the brains of patients suffering from Alzheimer’s disease (AD). Plaques are made up of β-amyloid peptides derived from amyloid precursor protein cleavage, and NFTs mainly contain paired helical filaments (PHFs), which are composed of hyperphosphorylated, fibrillar, microtubule-associated protein tau [1,2]. Hyperphosphorylated, fibrillar tau is also found in numerous neurodegenerative diseases that are collectively known as tauopathies, which include Picks disease, progressive supranuclear palsy, corticobasal degeneration and frontotemporal dementia (FTDP-17) [1]. Mutations in genes encoding for tau have been observed in the familial type of FTDP-17. These mutations result in tau hyperphosphorylation and fibrillization in experimental models both *in vivo* and *in vitro* [3,4,5,6,7,8,9,10,11]. In these tauopathies, neurodegeneration occurs in the absence of β-amyloid pathology [12]. Furthermore, studies using transgenic mice, primary neurons, and drosophila have shown that tau is required for β-amyloid neurotoxicity [13,14,15,16]. Tau dysfunction has been recognized as a central pathology in the development of AD. 

Tau is a neuron-specific microtubule-associated protein. In normal brain, it binds to and promotes the formation and stability of microtubules [2]. However, PHF-tau (tau isolated from PHFs) is hyperphosphorylated and does not bind to microtubules. Upon dephosphorylation, PHF-tau regains its microtubule-binding ability, suggesting that hyperphosphorylation prevents tau from associating with microtubules, leading to microtubule instability and eventual neurodegeneration in AD brain [17]. PHF-tau is phosphorylated at over 40 Ser/Thr sites [18,19,20]. In addition, it has also been reported that Tyr^18^, Tyr^197^ and Tyr^394^ are phosphorylated in PHFs [2]. Among these sites, Ser^256^, Ser^262^, Ser^289^ and Ser^356^ are located within the microtubule-binding region of tau [20]. The impact of phosphorylation at Ser^262^ has been studied the most, and phosphorylation at this site alone significantly reduces the affinity of tau for microtubules, and is sufficient in causing microtubule instability *in vitro* and *in vivo* [21]. 

In addition, in both primary neurons and drosophila, Ser^262^ tau phosphorylation mediates β-amyloid peptide toxicity in the brain [15,16]. Cdk5 and GSK3β are considered two of the main kinases that phosphorylate tau in AD brain [22,23,24,25,26,27,28]. In the drosophila model of tauopathy, tau Ser^262^ phosphorylation is a prerequisite for tau phosphorylation by Cdk5 and GSK3β [29]. Finally, synapse loss is regarded as the basis for dementia in AD patients [30]. Phosphorylation of tau at Ser^262^ causes a loss of pre- and postsynaptic proteins and reduces the number of dendritic spines in neurons [16]. These studies suggest that phosphorylation of tau at Ser^262^ plays an important role in the development of AD. *In vitro*, Ser^262^ is phosphorylated by a number of kinases including MARK, PKA, PKC, CamKII, and phosphorylase kinase [31,32,33,34]. How these kinases phosphorylate tau at Ser^262^ *in vivo* and in AD brain is not clearly understood. 

Understanding tau phosphorylation in the normal brain may provide insight into the mechanisms of abnormal tau phosphorylation in AD. Tau phosphorylation is developmentally regulated. In fetal brain cells that are still dividing, tau is highly phosphorylated [35]. As these cells differentiate into neurons and the brain develops into an adult state, tau phosphorylation, at many sites, becomes undetectable [35]. Interestingly, several of the same tau sites that are phosphorylated in AD are also phosphorylated in a normal, fetal brain during development [35,36,37,38,39]. It has been suggested that in the developing brain, tau phosphorylation is regulated by cell cycle mechanisms that reappear in AD brain [19,35,36,37,38,39]. Examining tau phosphorylation during brain development, therefore, may assist in determining the mechanism of tau hyperphosphorylation in AD. Neural development encompasses the differentiation of neurons from precursor cells. When dividing PC12 cells are exposed to Nerve Growth Factor (NGF), they slowly stop proliferating, develop neurites and growth cones, and differentiate into neurons. NGF-exposed PC12 cells are widely used to study neuronal differentiation [40,41,42,43,44]. 

To study how tau is phosphorylated during the transition of mitotic cells to terminally differentiated neurons, we have used NGF-exposed PC12 cells and found that adaptor protein 14-3-3ζ promotes tau phosphorylation at Ser^262^ by PKA in differentiating neurons. 14-3-3 are a family of proteins that regulate many different cellular functions including cell cycle, apoptosis, and signal transduction [45,46]. There are seven 14-3-3 isoforms in mammalian cells (β, ε, γ, η, σ, θ and ζ). Among these isoforms, 14-3-3ζ is involved in brain development and neuronal differentiation [47,48], and is upregulated in AD brain [49]. Therefore, to evaluate the pathological significance of our findings, we overexpressed 14-3-3ζ in rat primary neurons in culture. Herein, we report that overexpression of 14-3-3ζ promotes tau phosphorylation at Ser^262^ and causes proteosomal degradation of the presynaptic protein synaptophysin in primary neurons in culture. Loss of synaptophysin is recognized as an indicator of synaptic pathology in AD brain [30,50]. Our study suggests that 14-3-3ζ causes synaptic loss by destabilizing microtubules, leading to proteosomal degradation of synaptophysin in the neurons of patients suffering from AD.

## Materials and Methods

### Chemicals, Proteins and Antibodies

Olomoucine, forskolin, PKA inhibitor P9115 (the cell permeable myristoylated PKI inhibitor peptide 14-22), and PKAc (the catalytic subunit of PKA) were purchased from Sigma. Recombinant longest human tau isoform was purified from bacterial lysate [11]. Tau phosphorylation-specific antibodies used for the study were PHF-1 (1:1000; for phosphorylated Ser^396/404^), AT8 (1:500; for phosphorylated Ser^202/205^), 12E8 (1:500; for phosphorylated Ser^262^), pS214 (1:1000; for phosphorylated Ser^214^) and pS262 (1:1000; for phosphorylated Ser^262^), and were described previously [51]. AT180 (1:1000; for Thr^231^phosphorylated tau) was from Innogenetics. Monoclonal antibodies Tau 5 (1:1000; for total tau), anti-14-3-3ζ (1:500), anti-Myc (1:1000) and anti-Flag (1:1000) are also described previously [11,52]. Monoclonal antibody against synaptophysin (1:1000) was from Sigma. Anti-PSD-95 (1:500) and anti-N-cadherin (1:500) monoclonal antibodies were from Cell Signaling Technology Inc. Monoclonal anti-Ac-tubulin (1:250) and anti-Tyr-tubulin (1:250) were from Sigma. A polyclonal anti-synaptophysin (1:1000), and a monoclonal anti-active caspase 3 (1:200) were purchased from Millipore. Protein concentrations of each cell lysate were determined by the BioRad protein assay using BSA as the standard. Equal amounts of protein from each sample were separated on SDS-PAGE and then Western blotted. Primary antibody incubation (overnight) was followed by incubation with either rabbit polyclonal or mouse monoclonal secondary antibody linked to horseradish peroxidase (1:2000) for 4 hr. Immunoreactivity was visualized by using enhanced chemiluminescence reagent and exposure to X-ray film. Films were scanned densitometrically, and the optical density of bands was quantified using Image J software (NIH).

### Lentivirus Production, Neuronal Culture and Viral Infection

Human Myc-14-3-3ζ gene was subcloned into the pcDNA3 at Age1/Not1 sites using forward (5’- AAAAACCGGTATG GATAAAAATGAGCTG-3’) and reverse (5’-AAAAGCGGCCGCCTACAGATCTTCTTCAGAAATAAGTTTTTGTTCATTTTCCCCTCCTTCTCC-3’) primers. Myc-14-3-3ζ was subsequently subcloned into lentiviral vector pTet07 CSII-CMV-mcs-ires-GFPq, as described [51]. The viral DNA was transfected into 293SF-PacLV29-6 packaging cells. Virus was collected from the cell supernatant, concentrated, and its titer was determined. Hippocampal neurons were cultured from the E18 rat pups [51]. Neurons in culture for two weeks were infected with lentivirus (MOI of 10). After 48 hr of infection, neurons were lysed in lysis buffer (50 mM Tris-HCl (pH 7.4), 0.1 mM EDTA, 0.1 mM DTT, 150 mM NaCl, 50 mM β-glycerol phosphate, 0.1 mM EGTA, 10 mM MgCl_2_, 0.2% Nonidet P-40) and supplemented with phosphatase and protease inhibitor cocktail (Sigma), and subsequently analyzed. 

### cDNA Cloning, Cell Culture and Transfection

Murine PKA catalytic subunit (PKAc) and its dominant negative mutant PKA-DN (K272H) both in the pRSET_B_ vector (gifts of Dr. Susan Taylor of University of California, San Diego) were subcloned with a Myc tag into pcDNA3.1 by PCR at BamH1/ Nde1 site using forward 5’-ggt acc at ATG GGC AAC GCC GCC GCC GCC-3 and reverse 5’ - GGT ACC AAA CTC AGT AAA CTC CTT GCC -3’ primers. Dominant negative 14-3-3ζ (K49E) was a gift from Dr. Haian Fu of the Emory University, Atlanta, Georgia. PC12 cells were exposed to NGF (2.5 S) (0.1μg/ml) and their morphology was observed under the Nikon microscope as described [40,41]. In some experiments, PC12 cells were first transfected with the indicated cDNA for 6 hr using Lipofectamine 2000 and then NGF was added [41]. 

### Drug Treatments

PC12 cells exposed with NGF for six days were treated with P9115 (0.5 μM), EGTA (2 mM), LiCl (10 mM) or olomoucine (60 μM) for 1 hr, and harvested as described previously [41]. To inhibit lysosome, rat primary hippocampal neurons were infected with Ln-14-3-3ζ and were treated with bafilomycin (10 nM) or NH_4_Cl (10 mM) for 24 hr. To inhibit protein synthesis, infected neurons were treated with cycloheximide (10 μg/ml) for 2, 4, 8, 16 and 24 hr. To inhibit the proteosome, infected neurons were treated with MG132 (15 μM) or lactacystin (2.5 μM) for 24 hr as described [53]. 

### Immunocytochemistry

 Infected neurons were fixed with 4% paraformaldehyde in PBS at room temperature for 30 min. Fixed neurons were washed and permeabilized by incubating with PBS containing 0.1% Triton X-100 and 1% BSA for 30 min at room temperature. Neurons were then incubated for 12 hr at 4 °C with anti-synaptophysin mouse monoclonal (Sigma), 1:200 or rabbit polyclonal anti-Myc (Sigma-Aldrich), 1:200; washed and then labeled by incubating with Alexa Fluro 488 or Cy3 conjugated goat anti-mouse or goat anti-rabbit for 1 h at room temperature. 

### Image Acquisition and Quantification

 Images were obtained using Leica AF 6500 immunofluorescent microscope (Germany). Labeled transfected neurons were chosen randomly for quantification from 3-4 coverslips. The number of immunostained pucta were counted using MetaMorph Image analysis software (Universal Imaging Corporation). 

### *In vitro* Kinase Assay

PKA and Cdk5 activities were assayed as described previously using Kemptide (LRRASLG) and KTPKKAKKPKTPKKAKKI peptides, respectively [34]. In an assay mixture containing 50 mM Tris-HCl (pH 7.4), 1 mM DTT, 0.1 mM EDTA, 10 mM MgCl_2_, 0.5 mM [γ^32^P]ATP, the respective substrate peptides (20 μM), and phosphatase and protease inhibitor cocktails, an aliquot of cell lysate was added to initiate the reaction. After 30 min at 30 °C, the reaction was stopped by the addition of an equal volume of 20% TCA to each sample. Samples were placed on ice for 20 min and then centrifuged using a bench top centrifuge. The supernatant of each sample (20 μl) was analyzed using a filter paper assay to determine the amount of ^32^P incorporated into the peptide substrate [54]. 

### Quantitative Real Time PCR

 Total RNA was isolated from neurons infected with Ln-14-3-3ζ or Ln-vector using an RNeasy mini kit (Qiagen). Total RNA (1 μg) was used for the first-strand synthesis with reverse transcriptase II using oligodT or random primer (Invitrogen). SYBR^®^ Green based (Qiagen) real-time PCR was performed with gene specific primers for synaptophysin (Rn-Syp-2-SG) and GAPDH (Rn-GAPDH-1-SG) (Qiagen). Data was analyzed by Real-Time PCR software 7500 version 2.0.4 (Applied Biosystems). The relative RNA expression of the genes of interest were determined using the comparative ΔΔCt method with GAPDH as the endogenous control.

### Cell Survival

 MTT cell survival assay was carried out as described previously, using a kit from ATCC and following the manufacturer’s instruction manual [53]. Primary neurons in a 96-well plate were infected with Ln-14-3-3ζ or Ln-vector and incubated with MTT reagent at 37 °C in the dark. After 2 hr of incubation, the reaction was stopped and absorbance at 570 nm was monitored using an ELISA plate reader to quantify cell viability [53]. 

### Microtubule Sedimentation Assay

 A microtubule sedimentation assay was performed as described previously [55]. Neurons were lysed in Pipes buffer (0.1 M PIPES (pH 6.6), 1 mM EGTA, 1 mM MgSO_4_, 1 mM β-mercaptoethanol) containing 0.2% Nonidet P-40 as well as protease and phosphatase inhibitor cocktail. Each lysate was adjusted to 1 mg/ml protein concentration on ice and was transferred to a water bath at 37 °C. After 5 min, pre-warmed GTP (1 mM) and taxol (10 μM) were added to each sample. After 30 min of incubation at 37° C, samples were centrifuged at room temperature. The supernatant was designated as S. The pellet was gently washed with warm Pipes buffer containing GTP and taxol and was designated as P. Both S and P were analyzed by Western blot analysis. 

### Statistics

 The data is expressed as the mean ± SEM and was analyzed by one-way or two-way ANOVA followed by Bonferroni’s *post hoc* test for multigroup and the student’s t-test for two group comparisons. Differences with *p*<0.05 were considered significant. 

## Results

### Phosphorylation of Tau in NGF-Exposed PC12 Cells

PC12 cells did not display any obvious signs of differentiation during the first day of NGF exposure. After 1 day (24 hr) of NGF exposure, a few cells had developed protrusions. After 2 days (48 hr), ~50% cells had neurites of various lengths. By day 6, >90% cells were fully differentiated, displaying long neurites and growth cones (data not shown). 

**Figure 1:**
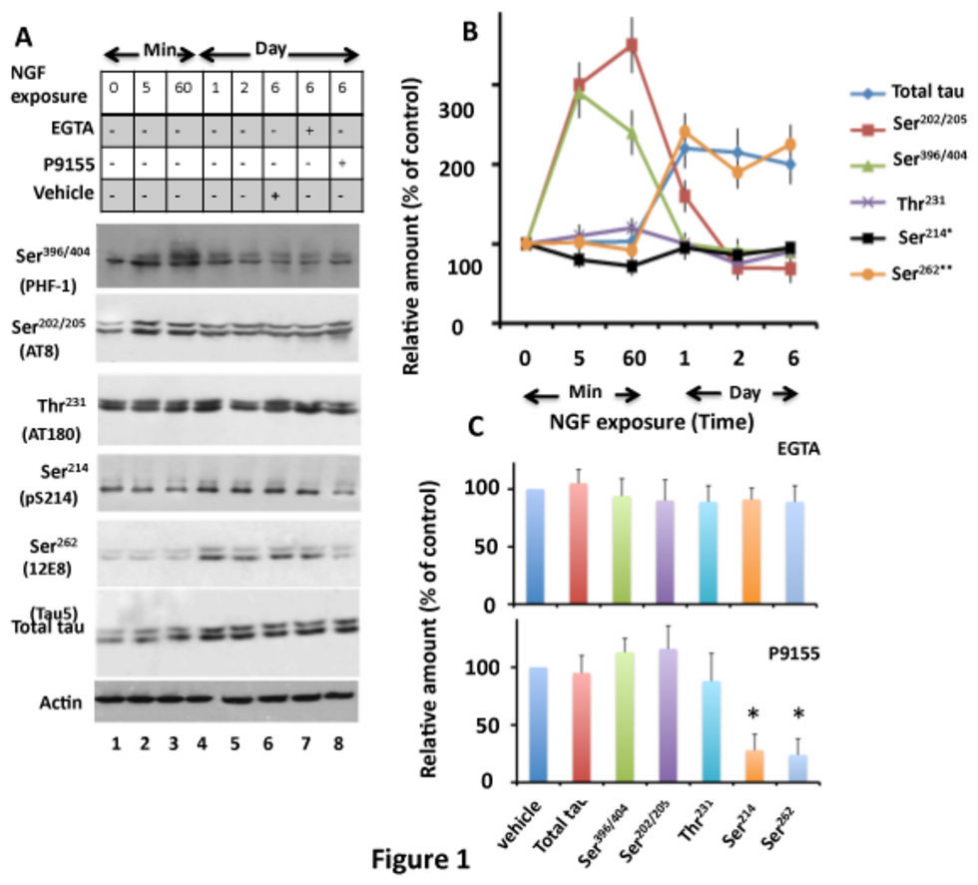
Phosphorylation of tau in NGF-exposed PC12 cells. PC12 cells exposed to NGF for the indicated time points followed by EGTA or P9115 were analyzed for tau phosphorylation by Western blot analysis. (A) Western blots. (B) Relative amount. The relative amount of phosphorylated tau at each site at the indicated time points was determined by normalizing the band intensity of phosphorylated tau with the respective tau band. The relative amount of total tau was determined by normalizing the tau band to the respective actin band. (C) Effects of EGTA and P9115. Band intensity values of tau or phosphorylated tau at indicated sites in cells exposed to NGF for 6 days and treated with EGTA (panel A, lane 7) or P9115 (panel A, lane 8) were normalized as in panel B and are expressed as the % of vehicle-treated control (panel A, lane 6). Values in panels B and C with standard error are the average of three determinations from three cultures. *p < 0.005 with respect to vehicle-treated cells.

To determine tau phosphorylation during PC12 cell differentiation, we probed NGF-exposed PC12 cell extracts with different antibodies that recognize tau phosphorylated site-specifically. As shown in Figure 1A, total tau level increased with NGF exposure time until day one (lanes 1-4), and then remained constant thereafter (lanes 4-6). Basal level of tau phosphorylation was observed in untreated cells (lane 1) and, upon NGF exposure, phosphorylation at all sites appeared to increase (lanes 2-6). However, when tau phosphorylation at each site was normalized against total tau, phosphorylation at proline-directed sites Ser^396/404^ and Ser^202/205^ were found to increase several fold within minutes of NGF exposure, but phosphorylation at Thr^231^ remained unchanged (Figure 1A, lanes 2-3). After one day of exposure, phosphorylation at both Ser^396/404^ and Ser^202/205^ sites decreased compared to the cells exposed for 60 min, but remained higher than the basal level (Figure 1A, lane 4 and B). After 2 and 6 days of NGF exposure, phosphorylation at both Ser^396/404^ and Ser^202/205^ sites remained comparable to that of 1 day of exposure. Phosphorylation at Thr^231^, however, remained unchanged throughout (Figure 1B).

Tau was phosphorylated in naive PC12 cells at the non-proline-directed sites Ser^214^ and Ser^262^ (Figure 1A, lane 1). Phosphorylation at Ser^214^ remained at basal level throughout the exposure to NGF (Figure 1A, lanes 2-6 and B). Ser^262^ phosphorylation (probed by 12E8 antibody), on the other hand, slowly increased during the initial phase (lanes 2-3), but surged to 2.2, 2.1 and 2.0 fold higher than the basal level on days 1, 2 and 6, respectively (Figure 1B). Similar observations were made when 12E8 antibody was replaced by pS262 antibody (data not shown). Thus, Ser^262^ phosphorylation became prominent at day 1 and remained at this level thereafter. As shown in Figure 1B, proline-directed Ser^396/404^ and Ser^202/205^ phosphorylation and non-proline-directed Ser^262^ phosphorylation in NGF exposed PC12 cells occurred sequentially. Proline-directed Ser^396/404^ and Ser^202/205^ phosphorylation occurred first, and as they began to decline, the non-proline-directed Ser^262^ phosphorylation began to increase, which peaked and was sustained at peak level. 

### Identification of Kinases

GSK3β is one of the major kinases that phosphorylates tau in the brain [25,26,27,56]. However, when NGF-exposed cells were treated with the GSK3β inhibitor LiCl (10 mM), tau phosphorylation at all of the examined sites including Ser^396/404^, Ser^202/205^, Thr^231^, Ser^214^ and Ser^262^ was similar when compared to the vehicle treated control cells (data not shown). This data is consistent with previous reports [57] and indicates that NGF does not activate GSK3β in PC12 cells. When LiCl was replaced by the Cdk5 inhibitor olmoucine [41], phosphorylation at both Ser^396/404^ and Ser^202/205^ was reduced by 78.9 and 69.5%, respectively, when compared to vehicle-treated controls (data not shown). This result is as expected and indicates that Cdk5 phosphorylates tau at both Ser^396/404^ and Ser^202/205^ sites in differentiated PC12 cells [51]. To identify the kinases responsible for non-proline-directed phosphorylation, we treated NGF-exposed PC12 cells with EGTA, a specific chelator of Ca^2+^, and analyzed them for tau protein phosphorylation (Figure 1A, lane 7). Phosphorylation at Ser^396/404^, Ser^202/205^, and Thr^231^ was similar in EGTA and vehicle treated cells (Figure 1C). Likewise, phosphorylation at Ser^214^ and Ser^262^ was also unaltered by EGTA (compare lanes 6 and 7 in Figure 1A and see Figure 1C)). Thus, depletion of Ca^2+^ did not inhibit tau phosphorylation at all of the examined sites. This result determined that Ca^2+^-dependent kinases PKC, CamKII, and phosphorylase kinase do not phosphorylate tau in NGF-exposed PC12 cells.

PKA phosphorylates tau *in vitro* and *in vivo* [33,58,59,60]. To determine if PKA was involved, we treated NGF-exposed PC12 cells with the cell permeable PKA inhibitor P9115. P9115 did not affect tau phosphorylation at Ser^396/404^, Ser^202/205^ or Thr^231^, but inhibited phosphorylation at Ser^214^ (Figures 1A, lane 8 and 1C), as expected [59]. Interestingly, P9115 also inhibited phosphorylation at Ser^262^ by ~76% (Figure 1C). To substantiate this result, we transfected PC12 cells with a dominant negative PKA mutant, Myc-PKA-DN, and exposed them to NGF (Figure S1). Tau phosphorylation at Ser^262^ and Ser^214^ were 42.1% and 65%, respectively less in Myc-PKA-DN transfected cells when compared to respective vector transfected controls (compare lane 2 with lane 3). Based on this result, we concluded that PKA phosphorylates tau at Ser^214^ and Ser^262^ in NGF-exposed PC12 cells.

### NGF Does Not Activate PKA in PC12 Cells

**Figure 2:**
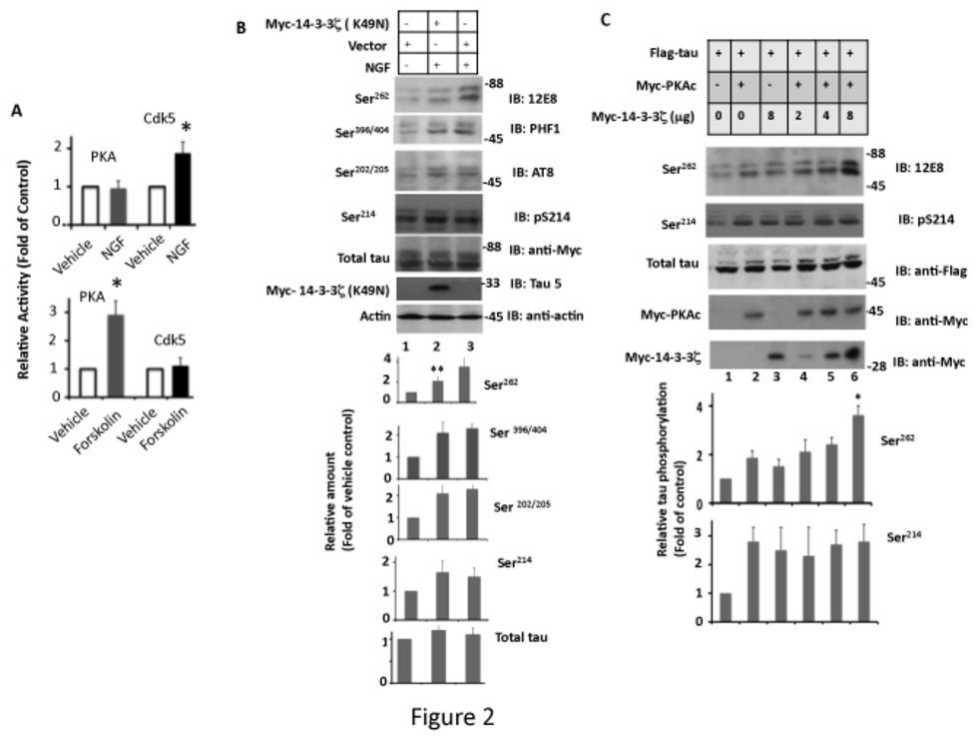
14-3-3ζ promotes PKA-catalyzed Ser^262^ tau phosphorylation in PC12 and HEK-293 cells. (A) NGF does not activate PKA in PC12 cells. PC12 cells were treated with forskolin, NGF, or vehicle for 24 hr. Treated cells were lysed and each lysate was assayed for PKA or Cdk5 activity using the respective peptide substrate. Values are the average of three determinations from three cultures, and are expressed as the fold change from vehicle-treated control cells. *p<0.005 with respect to vehicle treated cells. (B) Disruption of 14-3-3ζ function inhibits tau phosphorylation at Ser^262^ in NGF-exposed PC12 cells. PC12 cells transfected with Myc-14-3-3ζ (K49N) or empty vector were exposed to NGF for 24 hr and then analyzed by Western blotting as in Figure 1. Values with standard error are the average of three determinations from three cultures. **p < 0.001 with respect to vector transfected and NGF-treated cells. (C) 14-3-3ζ promotes PKA-catalyzed tau Ser^262^ phosphorylation in HEK293 cells. HEK293 cells co-transfected with Flag-tau and Myc-14-3-3ζ were analyzed by Western blotting. The relative amount of Ser^262^ phosphorylated tau was determined by normalizing the Ser^262^ band in each lane to the respective Flag-tau band. Values with standard error are the average of three determinations from three cultures. *p < 0.005 with respect to cells transfected with Flag-tau and Myc-PKAc.

PKA exists as an inactive dimer, composed of a catalytic PKAc subunit and an inhibitory subunit. Dissociation of the inhibitory subunit from the holoenzyme via cAMP signaling activates PKAc [61]. To test if NGF activates PKA, we measured PKA activity by an *in vitro* kinase assay in PC12 cell extract exposed to NGF or the PKA agonist forskolin. Forskolin activated PKA but not Cdk5, as expected (Figure 2A, lower panel). However, NGF activated Cdk5 but had no effect on PKA activity (Figure 2A, upper panel). This result determined that NGF does not activate PKA in PC12 cells. 

### 14-3-3ζ is Involved in Tau Phosphorylation in Mammalian Cells

Previous studies have reported that, *in vitro*, PKA phosphorylates tau at Ser^262^ [31,33] and that, 14-3-3ζ binds to tau and promotes Ser^262^ phosphorylation by PKA [62]. Although a rise in intracellular cAMP level activates PKA *in vivo*, a basal PKA activity has been observed in a number of cell lines including PC12 cells [63,64,65]. Furthermore, when PC12 cells are exposed to NGF, 14-3-3ζ expression significantly increases [66]. NGF, therefore, may promote tau phosphorylation via basal PKA activity at Ser^262^ indirectly by enhancing 14-3-3ζ level in cells. To test this idea, we transfected PC12 cells with a dominant negative 14-3-3ζ mutant Myc-14-3-3ζ (K49N) and then exposed them to NGF. Myc-14-3-3ζ (K49N) inhibited NGF-induced Ser^262^ phosphorylation by 40% but did not affect phosphorylation at Ser^214^, Ser^396/404^ or Ser^202/205^ (Figure 2B, lane 2). To substantiate this result, we transfected HEK-293 cells with Flag-tau, Myc-PKAc, and different amounts of Myc-14-3-3ζ, and analyzed tau phosphorylation at Ser^262^ and Ser^214^. PKA phosphorylated tau at both Ser^214^ and Ser^262^. However, Myc-14-3-3ζ promoted tau phosphorylation at Ser^262^ but not at Ser^214^ (Figure 2C, lanes 4-6). Similar observations were made when pS262 antibody was used (data not shown). Based on these data, we concluded that 14-3-3ζ promotes tau Ser^262^ phosphorylation in intact cells.

### Overexpression of 14-3-3ζ Promotes Tau Phosphorylation at Ser^262^ and Inhibits Tau Microtubule Binding in Rat Primary Neurons in Culture

Ser^262^ is phosphorylated in PHF-tau [18,67], and phosphorylation at this site causes microtubule instability and neurodegeneration in models both *in vitro* and *in vivo* [15,16,21,29,55]. 14-3-3ζ, on the other hand, was reported to be upregulated in AD brain and localized in neurons with high NFT densities [49,68,69]. We found that 14-3-3ζ promotes tau Ser^262^ phosphorylation during neuronal differentiation (Figure 1). To evaluate the pathophysiological significance of these observations, we infected rat primary hippocampal neurons with lentivirus expressing human Myc-14-3-3ζ (Ln-14-3-3ζ) and analyzed them for tau phosphorylation. 

**Figure 3:**
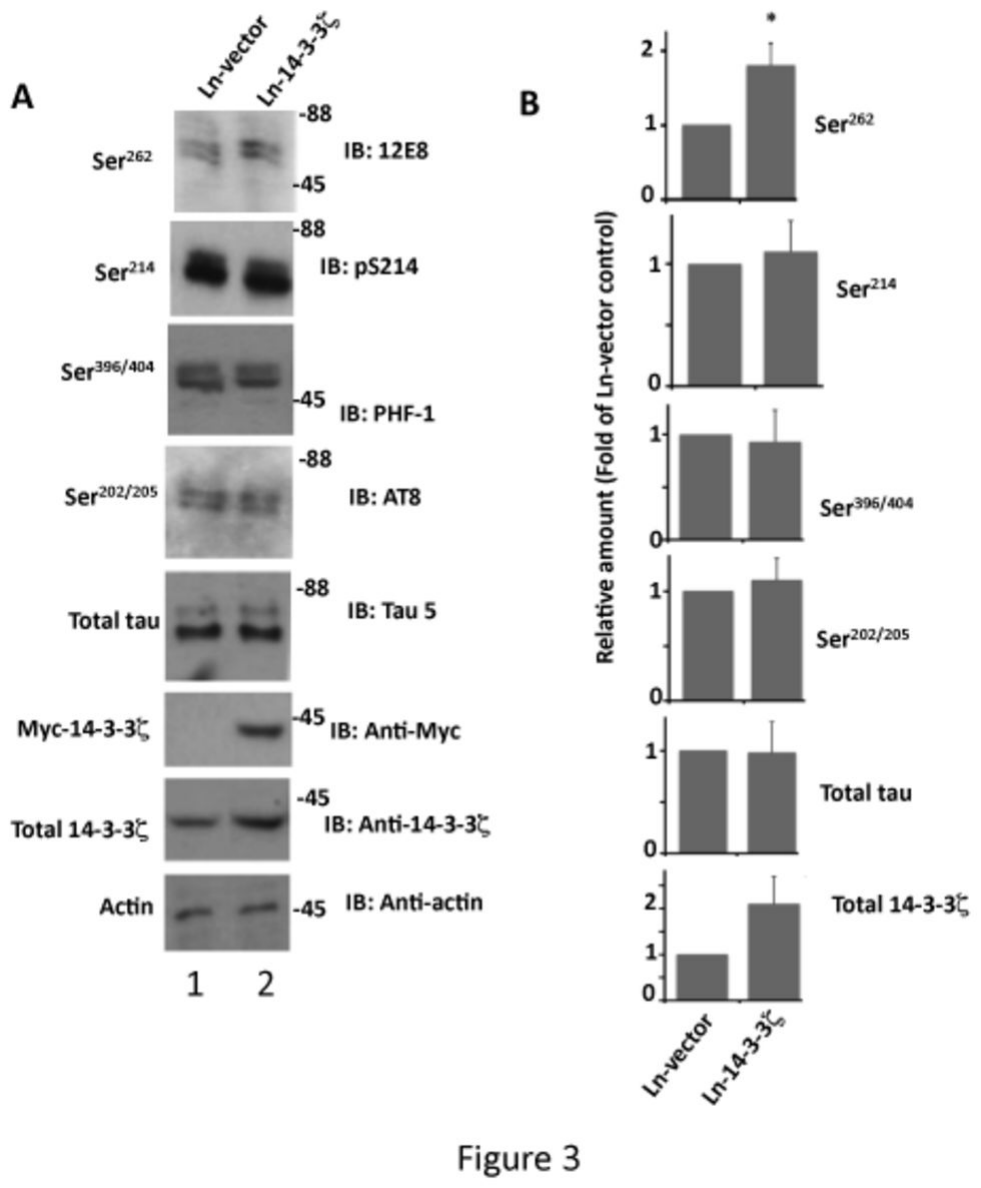
Overexpression of 14-3-3ζ promotes tau phosphorylation at Ser^262^ in rat hippocampal primary neurons in culture. Rat hippocampal primary neurons in culture infected with Ln-14-3-3ζ or Ln-vector were analyzed for tau phosphorylation by Western blotting as in Figure 1. (A) Western blots, (B) Relative amounts. Values with standard error are the average of three determinations from three cultures. *p < 0.001 with respect to Ln-vector infected neurons.

 14-3-3ζ overexpression did not change tau levels and did not affect tau phosphorylation at Ser^396/404^, Ser^202/205^ or Ser^214^ significantly, but caused increased 12E8 immunoreactivity by ~1.8-fold (Figure 3A, B) and pS262 immunoreactivity by 2.1-fold (data not shown). Thus, as observed in HEK-293 cells, overexpression of 14-3-3ζ promotes tau phosphorylation at Ser^262^ in neurons.

 In neurons, tau binds to microtubules and promotes microtubule polymerization. Ser^262^ phosphorylation significantly affects the tau-microtubule interaction *in vitro* [21]. Since 14-3-3ζ promotes tau phosphorylation at this site, it is possible that 14-3-3ζ overexpression may affect tau microtubule binding. To examine this possibility, we performed a microtubule sedimentation assay using neurons infected with Ln-14-3-3ζ and Ln-vector. 

**Figure 4:**
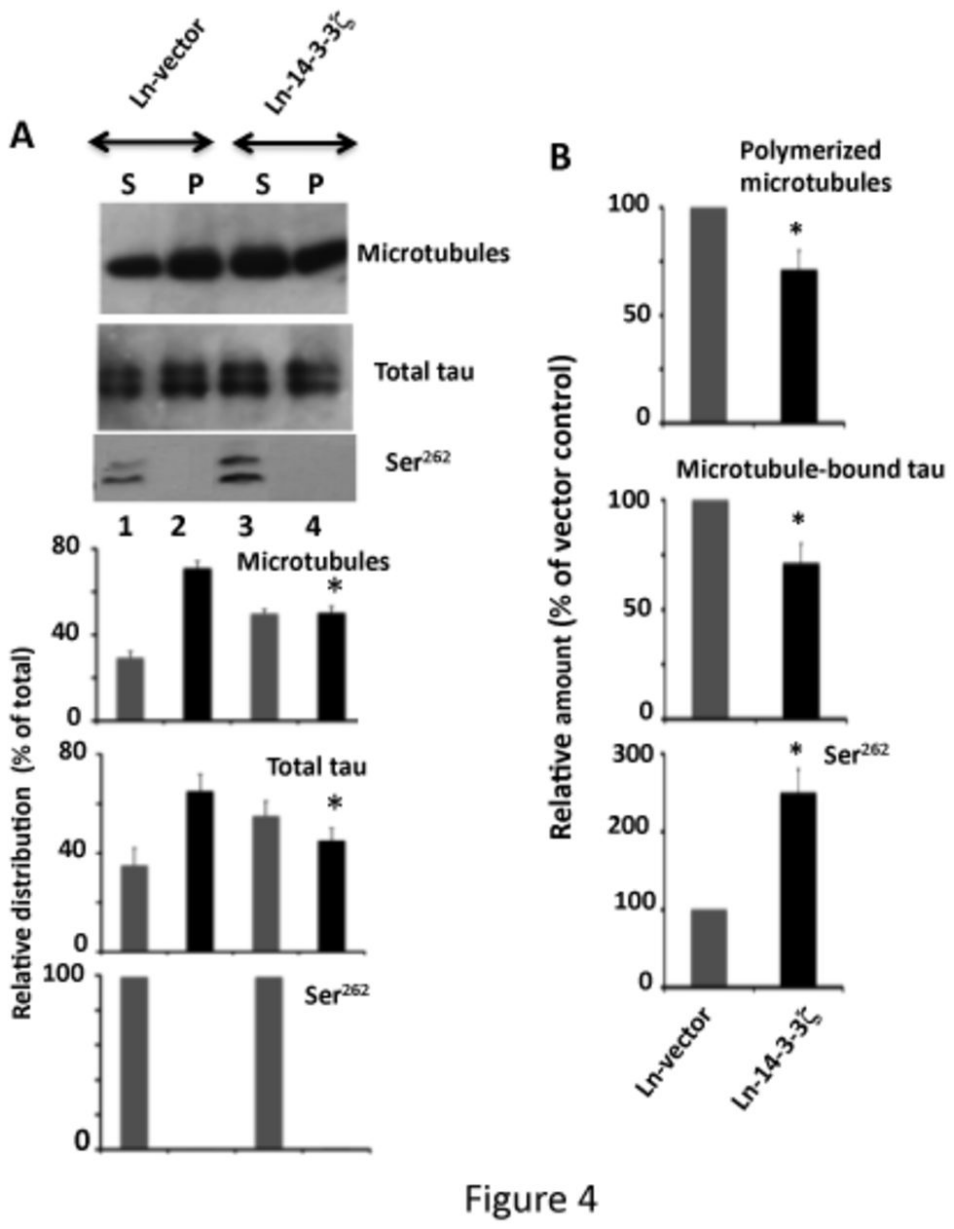
Overexpression of 14-3-3ζ inhibits tau binding to microtubules and reduces the amount of polymerized microtubules in rat hippocampal primary neurons in culture. Primary neurons infected with Ln-14-3-3ζ or Ln-vector were subjected to a microtubule sedimentation assay. The resulting microtubule pellet (P) and the supernatant (S) were analyzed by Western blotting and the relative distribution of each protein in its respective fraction was determined. The relative distribution value was determined by dividing the band intensity of a protein in the fraction by the total (sum of the intensity value of that protein, both S and P fractions), and is expressed as a % of the total. Values with S.E. are an average of three determinations from three cultures. *p< 0.05 with respect to the P fraction of the Ln-vector control. (B) Relative amount. The relative amounts of polymerized microtubules are relative distribution values from the microtubule pellet in panel (A) and are expressed as the % of Ln-vector control. Likewise, the relative amounts of microtubule-bound tau are the values of total tau in microtubule pellet in panel (A), and are expressed as a % of Ln-vector control. The relative amount of Ser^262^ phosphorylated tau was determined by normalizing the Ser^262^ blot by the corresponding tau blot, as in Figure 1. Values are average of three determinations from three cultures. *p<0.05 with respect to the Ln-vector control.

 As shown in Figure 4A, in Ln-vector infected neurons, 70.7% of the total tubulin formed microtubules and settled in the pellet (P) (lane 2). In 14-3-3ζ infected neurons, on the other hand, 50.2% of the total tubulin formed microtubules (lane 4). Thus, compared to Ln-vector infected neurons, Ln-14-3-3ζ infected neurons had a 29.1% reduction in the amount of polymerized microtubules (Figure 4B). In Ln-vector infected control neurons, 65% of the total tau bound to microtubules and settled in the microtubule pellet (Figure 4B, lane 2). In Ln-14-3-3ζ infected neurons, however, 45% of the total tau was microtubule bound (lane 4), a reduction of 30.7% compared to Ln-vector infected neurons (Figure 4B). 

The relative amount of Ser^262^ phosphorylated tau was 2.8-fold more in Ln-14-3-3ζ infected neurons than in Ln-vector infected (Figure 4B). However, in all neurons Ser^262^ phosphorylated tau was exclusively in the supernatant (Figure 4A, lanes 1 and 3) and was undetectable in the microtubule pellet (Figure 4A, lanes 2 and 4). This result indicates that Ser^262^ phosphorylated tau does not bind to microtubules. Thus, 14-3-3ζ overexpression promotes tau phosphorylation at Ser^262^, inhibits tau microtubule binding, and reduces the amount of polymerized microtubules in the neurons.

### 14-3-3ζ Overexpression Destabilizes Microtubules in Rat Primary Neurons in Culture

**Figure 5:**
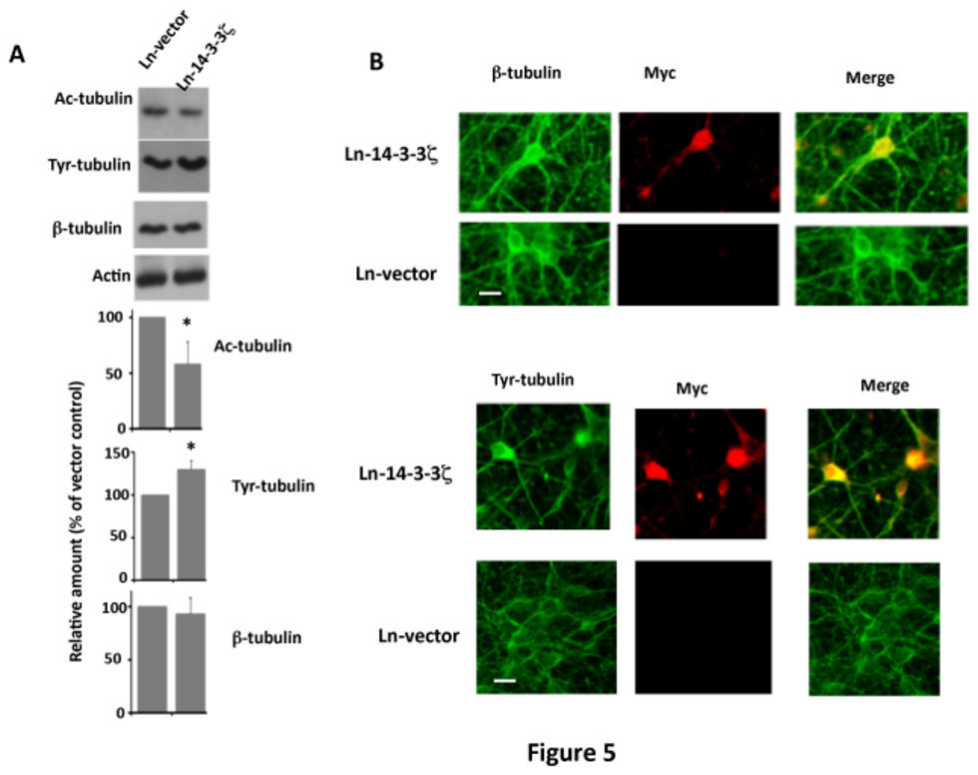
Overexpression of 14-3-3ζ in primary neurons in culture causes microtubule instability. Neurons infected with Ln-14-3-3ζ or Ln-vector were analyzed for microtubule instability by Western blotting or immunocytochemistry. (A) Western blot analysis. Western blot analysis for Ac-tubulin (stable microtubules), Tyr-tubulin (unstable microtubules) or β-tubulin (total tubulin) was performed. The Ac-tubulin or Tyr-tubulin band of each sample was normalized against the respective total tubulin band to determine the corresponding relative amount. To determine the relative amount of total tubulin, the tubulin band was normalized against the respective actin band. Values with standard error are the average of three determinations from three cultures. *p< 0.05 with respect to Ln-vector infected controls. (B) Immunocytochemistry. Representative immunofluorescence micrographs of infected neurons immunostained with anti-β-tubulin (total tubulin), anti-Myc (Myc-14-3-3ζ), or anti-Tyr-tubulin. Scale bar. 25 μm.

Tau binds to and promotes the stability of microtubules *in vitro* [2,11]. Since 14-3-3ζ overexpression inhibited tau microtubule binding (Figure 4), we examined microtubule stability in neurons infected with Ln-14-3-3ζ. 14-3-3ζ overexpression did not affect tubulin turnover, as the level of tubulin was similar in 14-3-3ζ and vector overexpressing neurons (Figure 5A). However, compared to Ln-vector infected neurons, the relative amount of stable Ac-tubulin was 42% less in 14-3-3ζ expressing neurons (Figure 5A). Likewise, the relative amount of Tyr-tubulin representing unstable microtubules was 30% more in 14-3-3ζ overexpressing neurons than in those expressing vector (Figure 4A). Under the fluorescent microscope, Ln-vector infected neurons had a relatively weak Tyr tubulin signal in the cell body and in neurites. In Ln-14-3-3ζ infected neurons, on the other hand, there was an intense Tyr-tubulin signal in the cell body and in hillocks (Figure 5B, lower). Total tubulin immunostaining was similar in both Ln-14-3-3ζ and Ln-vector infected neurons. These data determined that 14-3-3ζ overexpression destabilizes the microtubule cytoskeleton in rat hippocampal primary neurons in culture. 

### Overexpression of 14-3-3ζ Downregulates Synaptophysin Protein Level in Neurons

Previous studies have shown that tau phosphorylated at Ser^262^ promotes neurodegeneration [29]. 14-3-3ζ enhances Ser^262^ phosphorylation in neurons (Figure 3). Therefore, to evaluate the pathological impact of 14-3-3ζ overexpression, we first performed an MTT cell survival assay of neurons infected with either Ln-vector or Ln-14-3-3ζ. The % of live cells in 14-3-3ζ expressing neurons was similar (105.1 ± 15) to those expressing vector control (Figure S2A). When Western blotted for active caspase 3, the respective bands resulting from 14-3-3ζ and vector expressing neuronal lysates were barely detectable (Figure S2B). Thus, overexpression of 14-3-3ζ did not affect cell survival and did not promote apoptosis in neurons. 

**Figure 6:**
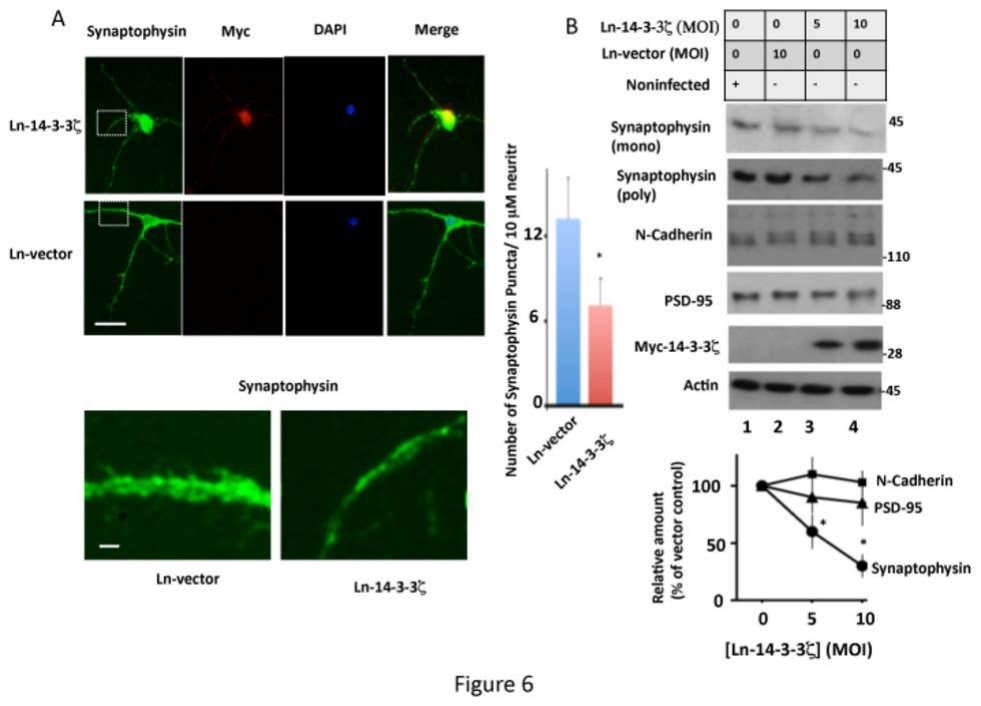
Overexpression of 14-3-3ζ downregulates synaptophysin protein level in rat hippocampal primary neurons in culture – Neurons infected with Ln-14-3-3ζ or Ln-vector were analyzed for synaptophysin protein levels by immunocytochemistry or Western blotting. (A) Immunocytochemistry. Representative immunofluorescent micrographs are of neurons infected with the indicated virus and stained for Myc (14-3-3ζ), synaptophysin, DAPI (nucleus), and merge (co-localization). The corresponding inset is shown in higher magnification in the lower panel. Quantification of synaptophysin puncta from 50 neurites in each group from three different cultures is shown on the right hand side panel. Scale bars: upper 15 μm; lower, 5 μm. *p<0.005 with respect to Ln-vector infected neurons. (B) Western blot analysis. Representative Western blots from extracts of neurons infected with Ln-14-3-3ζ or Ln-vector showing the level of indicated synaptic proteins in each culture. The relative amount of each protein was determined from the blots by normalizing the band intensity of that protein against the respective actin band. Values with standard error are an average of three determinations from three cultures. *p< 0.05 with respect to Ln-vector infected cells.

14-3-3ζ is present in synapses [46], and synapse loss precedes neurodegeneration in AD [70]. Primary neurons in culture develop synapses similar to those observed in the CNS, and neurons that are 3 weeks in culture have fully formed synapses. Synaptophysin protein level is widely used as a synaptic marker [71]. Therefore, we evaluated the effect of 14-3-3ζ overexpression on synapses by measuring synaptophysin protein level (Figure 6). Ln-vector infected neurons displayed numerous synaptophysin clusters in the cell soma and neurites, as expected (Figure 6A). In Ln-14-3-3ζ infected neurons, however, the number of labeled synaptophysin clusters was 52% less than in those expressing Ln-vector. In addition, in Ln-14-3-3ζ infected neurons, the synaptophysin clusters were less intense and relatively smaller in size when compared with those infected with Ln-vector. 

Western blot analysis determined that, compared to Ln-vector infected neurons, the intensity of the synaptophysin protein band was significantly less in Ln-14-3-3ζ-infected neurons (compare lane 2 with lanes 3 and 4 in Figure 6B). Similar observations were made when another anti-synaptophysin antibody was used (Figure 6B, upper panel). 14-3-3ζ overexpression did not, however, affect the levels of post-synaptic marker protein, PSD-95, or the synaptic adhesion protein N-cadherin, in neurons (Figure 6B). Based on this result, we concluded that 14-3-3ζ overexpression specifically downregulates synaptophysin protein level in rat hippocampal primary neurons in culture. 

### 14-3-3ζ Overexpression Promotes Proteosomal Degradation of Synaptophysin in Neurons

To determine if 14-3-3ζ overexpression suppresses synaptophysin transcription, we isolated mRNA from Ln-vector and Ln-14-3-3ζ -infected neurons and performed quantitative real-time PCR as described in Materials and Methods. The level of synaptophysin mRNA in Ln-14-3-3ζ infected neurons was similar (88% ± 20) to that observed in Ln-vector-infected neurons (Figure S3). This result indicated that 14-3-3ζ overexpression does not affect synaptophysin gene expression in rat primary neurons.

**Figure 7:**
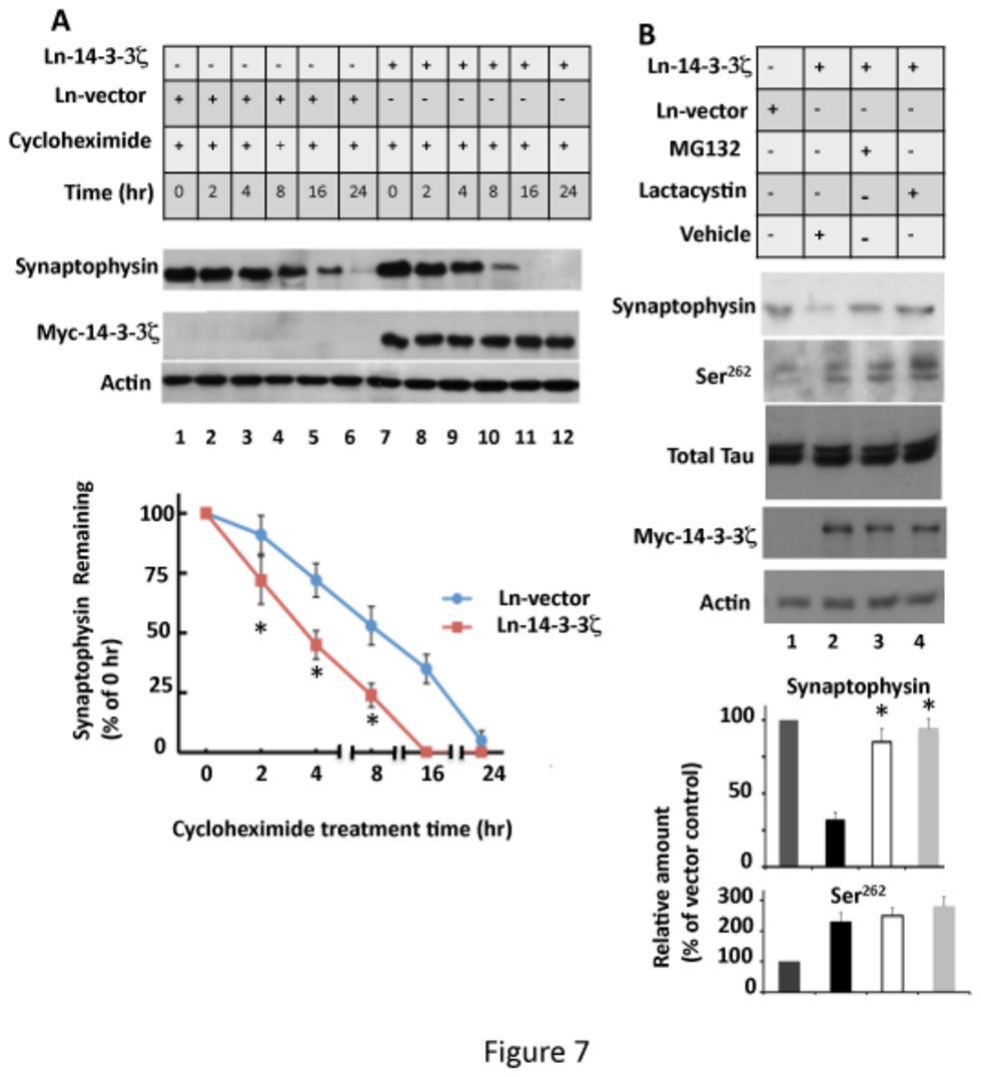
Overexpression of 14-3-3ζ in rat primary hippocampal neurons promotes proteosomal degradation of synaptophysin protein (A) 14-3-3ζ overexpression does not affect synaptophysin protein synthesis in neurons. Ln-14-3-3ζ or Ln-vector infected neurons were treated with cycloheximide or vehicle for the indicated time and analyzed for synaptophysin protein by Western blotting. Synaptophysin band intensity in each lane was normalized against the corresponding actin band and is expressed as the % of 0 hr. Values with ± SE are the average of three determinations. *p<0.005 with respect to Ln-vector infected neurons. (B) Proteosome inhibitors block the downregulation of synaptophysin protein caused by 14-3-3ζ overexpression. Primary neurons infected with Ln-14-3-3ζ or Ln-vector were treated with MG132 or lactacystin for 24 hr. Treated neurons were analyzed by Western blotting and the relative amounts were determined as per Figure 6. Values with standard error are the average of three determinations from three cultures. *p< 0.005 with respect to Ln-14-3-3ζ infected and vehicle treated neurons.

In primary neurons, synaptophysin level is controlled both by mRNA translation and protein degradation [72,73]. To determine by which of the above mechanisms 14-3-3ζ down regulates synaptophysin level, we treated 14-3-3ζ-infected neurons with the protein synthesis inhibitor cycloheximide and monitored synaptophysin protein levels. As shown in Figure 7A, in Ln-vector infected neurons, synaptophysin level progressively decreased with an increase in post-drug treatment time (lanes 1-6) and became almost undetectable in 24 hr (lane 6). In 14-3-3ζ overexpressing neurons, synaptophysin level disappeared faster than in Ln-vector infected neurons (Figure 7A, lower). At the 2 hr time point, 28% of the total synaptophysin was degraded in 14-3-3ζ overexpressing neurons, whereas only 9% was degraded in the Ln-vector infected neurons. At the 4 and 8 hr time points, this value increased to 55 and 76%, respectively, in 14-3-3ζ expressing neurons, and 28 and 47%, respectively, in the vector expressing neurons. Thus, despite the blockage of protein synthesis, synaptophysin turnover continued to occur in both vector and 14-3-3ζ infected neurons. More importantly, in 14-3-3ζ expressing neurons, synaptophysin turnover was significantly faster than in vector expressing neurons (Figure 7A, lower). This result determined that 14-3-3ζ overexpression accelerated synaptophysin protein degradation in primary neurons in culture. 

Lysosome and proteosome are the two major intracellular protein degrading systems in eukaryotic cells. To determine which of the two systems is involved in synaptophysin degradation, we treated Ln-14-3-3ζ infected neurons with lysosome inhibitor bafilomycin or NH_4_Cl (Figure S4). The level of synaptophysin protein in 14-3-3ζ-infected and bafinomycin treated neurons was similar to those infected with Ln-14-3-3ζ and treated with vehicle (compare lane 2 with lane 4) or NH_4_Cl (compare lane 2 with lane 6). This result excluded the involvement of lysosomes. 

To evaluate if the proteosome is involved, we treated neurons overexpressing 14-3-3ζ with the proteosome inhibitor MG132 or lactacystin [53]. MG132 (carbobenzoxy-Leu-Leu-leucinal) is a peptide aldehyde that effectively blocks the proteolytic activity of the 26S proteosome complex. Lactacystin is structurally different from MG132 and is more specific, inhibiting proteosome function by acting as a pseudosubstrate and becoming covalently linked to the active site of the 20S subunit [74]. In neurons infected with Ln-14-3-3ζ and treated with MG132, the synaptophysin protein level was 2.6-fold more than in those infected with Ln-14-3-3ζ and treated with vehicle (Figure 7B, lane 3). Likewise, lactacystin treatment increased synaptophysin protein level in 14-3-3ζ expressing neurons by 2.9-fold (Figure 7B, lane 4). Based on these data, we concluded that overexpression of 14-3-3ζ promotes proteosomal degradation of synaptophysin in rat hippocampal primary neurons in culture. 

### 14-3-3ζ Promotes Proteosomal Degradation of Synaptophysin by Destabilizing Microtubules in Neurons

Tau phosphorylation activates the proteosome in HEK-293 cells [75], and microtubule disruption leads to enhanced proteosomal activity in neurons [76]. 14-3-3ζ promotes tau phosphorylation at Ser^262^ and destabilizes microtubules (Figures 3-5). Therefore, it is possible that 14-3-3ζ promotes proteosomal degradation of synaptophysin by destabilizing microtubules. To test this possibility, we treated Ln-14-3-3ζ infected neurons with the microtubule stabilizing drug taxol, and then analyzed them. 

**Figure 8:**
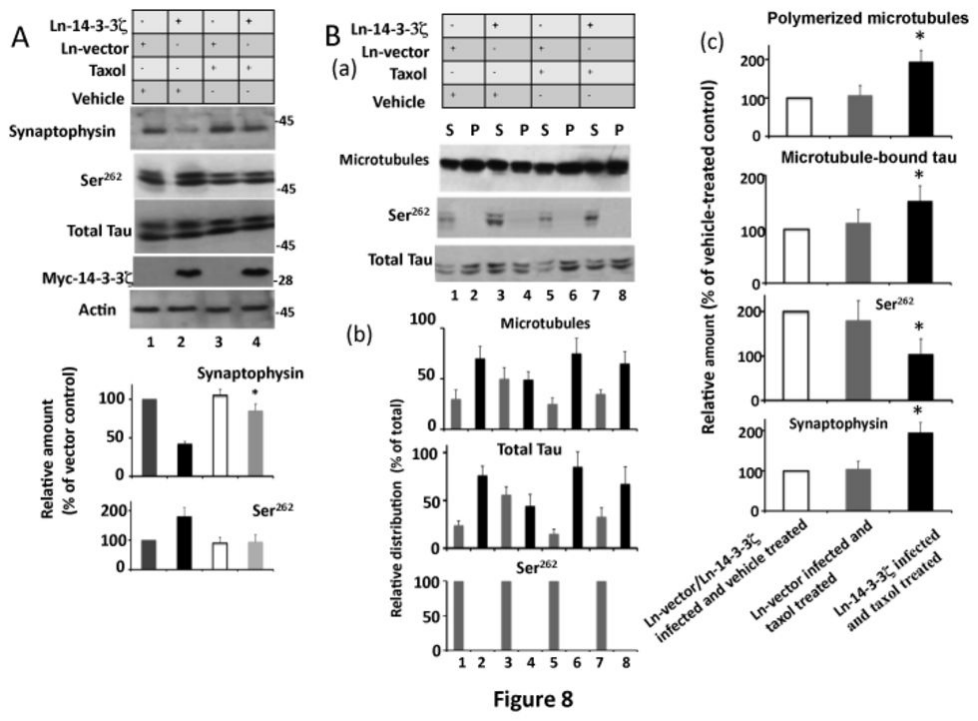
Microtubule stabilizing drug taxol restores synaptophysin protein levels in 14-3-3ζ overexpressing neurons. Neurons infected with Ln-14-3-3ζ or Ln-vector were treated with the microtubule stabilizing drug taxol for 24 hr and then analyzed by Western blotting to determine the relative amounts, and then subjected to a microtubule sedimentation assay. (A) Western blot analysis. The relative amount of each protein shown in the lower panel was determined as per Figure 5. Data with standard error are the average of three determinations from three cultures. *p < 00.1 with respect to 14-3-3ζ infected and vehicle treated neurons. (B) Microtubule sedimentation assay. The microtubule sedimentation assay was performed as in Figure 4. The resulting microtubule pellet (P) and the supernatant (S) were analyzed by Western blotting and the relative distribution and relative amounts were determined as in Figure 4. (a) Western blots. (b), relative distribution. Values with S.E. are average of three determinations from three cultures. *p< 0.05 with respect to the P fraction of Ln-vector control. (c) Relative amounts. The relative amount of polymerized microtubules are relative distribution values from the microtubule pellet in panel (b) and are expressed as the % of Ln-vector control. Likewise, the relative amounts of microtubule-bound tau are the values of total tau in the microtubule pellet in panel (b) and are expressed as a % of Ln-vector control. The relative amount of Ser^262^ phosphorylated tau was determined by normalizing Ser^262^ blot by corresponding tau blot as in Figure 4. Values are an average of three determinations from three cultures. *p<0.05 with respect to Ln-vector control.

Western blot analysis showed that synaptophysin levels were reduced in Ln-14-3-3ζ-infected neurons (Figure 8A, lane 2). Taxol treated neurons that were infected with Ln-14-3-3ζ increased synaptophysin protein levels 2.1-fold compared to vehicle-treated controls (Figure 8A, lane 4). In addition, taxol reduced tau phosphorylation at Ser^262^ by 2.1-fold in Ln-14-3-3ζ infected neurons (lane 4). Thus, taxol treatment reduced tau phosphorylation at Ser^262^ and provided significant protection against downregulation of synaptophysin induced by 14-3-3ζ overexpression. 

To get more insight into the effects of taxol in Ln-14-3-3ζ overexpressing neurons, we performed a microtubule sedimentation assay. As shown in Figure 8B, compared to Ln-vector infected and vehicle-treated neurons, Ln-14-3-3ζ infected and vehicle-treated neurons had significantly lower amounts of polymerized microtubules in the pellet (compare lane 4 with lane 2). Similarly, the amount of tau in the microtubule pellet was significantly less in Ln-14-3-3ζ infected and vehicle-treated neurons than in those infected with Ln-vector and treated with vehicle (compare lane 4 with lane 2). This observation is consistent with Figure 4 data and showed that 14-3-3ζ overexpression reduces the amount of microtubule-bound tau as well as the amount of polymerized microtubules in neurons.

More importantly, the amount of polymerized microtubules, microtubule-bound tau, Ser^262^ phosphorylated tau, and synaptophysin were similar in Ln-vector infected and vehicle-treated as well as Ln-vector infected and taxol treated neurons (Figure 8B (c)). However, compared to Ln-14-3-3ζ infected and vehicle treated neurons, Ln-14-3-3ζ infected and taxol treated neurons had almost 2-fold more polymerized microtubules, and microtubule-bound tau was 1.5 fold higher. This result indicates that taxol also protects neurons from 14-3-3ζ-induced loss of polymerized microtubules and reduction in the amount of microtubule bound tau.

## Discussion

Tau is phosphorylated at multiple Ser/Thr sites which are divided into two classes: proline-directed and non proline-directed [35]. Proline-directed sites are phosphorylated by proline-directed kinases such as Cdk1, Cdk2, MAPK, Cdk5 and GSK3β. Non proline-directed sites are phosphorylated by PKA, PKC, CamKII, MARK, and phosphorylase kinase [25,62]. These kinases are activated by different stimuli and regulate different cellular functions. Although tau phosphorylation has mainly been implicated to regulate microtubule dynamics, current studies suggest that it is also involved in signal transduction [77]. 

In proliferating PC12 cells, tau is phosphorylated on all sites examined (Figure 1A, lane 1). When these cells are treated with NGF, phosphorylation at proline-directed sites Ser^396/404^ and Ser^202/205^ increases first (Figure 1). During this period, kinases are activated which then phosphorylate their respective cytosolic and nuclear substrates, and the NGF-induced signal propagates from the cell surface to the cytosol. Consequently, cells commit to differentiation [43]. With increasing time of NGF exposure, tau phosphorylation at proline-directed sites declines but is maintained at basal level, and non-proline-directed Ser^262^ phosphorylation becomes more and more prominent (Figure 1). During this period, neurites are formed and extended [78]. Neurite formation and extension requires the assembly of microtubules that run longitudinally within the neurite shaft [40,78]. Our data suggest that during neuronal differentiation, proline-directed tau phosphorylation is involved in signal propagation from the cell surface. Phosphorylation at Ser^262^, on the other hand, regulates microtubule dynamics during neurite outgrowth and growth cone formation. 

When NGF-exposed PC12 cells are treated with the PKA inhibitor P9115 or transfected with PKA-DN, Ser^262^ phosphorylation is significantly inhibited (Figures 1, 2). This data indicates that NGF promotes Ser^262^ phosphorylation by PKA. Surprisingly however, NGF does not activate PKA in these cells (Figure 2A). Previous studies have shown that PC12 cells have a basal level of PKA activity [63], and that when these cells are treated with NGF, expression of 14-3-3ζ is increased many fold, activating 14-3-3ζ-dependent events [66]. 14-3-3ζ is an adapter protein that regulates the function of its target proteins by binding to them [45,46]. *In vitro*, 14-3-3ζ binds to tau and promotes PKA-catalyzed tau Ser^262^ phosphorylation [62]. These observations and our data together suggest that NGF promotes Ser^262^ phosphorylation of tau by PKA in PC12 cells via enhancing intracellular 14-3-3ζ levels. 

Abnormal tau phosphorylation is suggested to be fundamental to the development of NFT pathology in AD [1,2,17]. Although tau is phosphorylated at a number of sites, current studies indicate that phosphorylation at Ser^262^ has a higher pathological impact. In mouse brain and drosophila, both β-amyloid peptide and DNA damage cause neurodegeneration by promoting Ser^262^ phosphorylation [15,29]. In rat primary neurons in culture, accumulation of Ser^262^ phosphorylated tau causes synapse loss that is evident by the reduction of pre- and postsynaptic proteins [16]. The proteoglycan heparin, implicated to cause tau fibrillization in AD brain [79], promotes tau phosphorylation at Ser^262^ in vitro [54]. Neurotoxin MPTP promotes tau phosphorylation at Ser^262^ in human neuroblastoma cells [55]. Likewise α-synuclein, a component of senile plaques [80], promotes tau phosphorylation at Ser^262^ [55]. Sporadic AD is suggested to be multifactorial [1]. As discussed above, diverse factors that are implicated in causing AD pathology act via promotion of tau phosphorylation at Ser^262^. These observations highlight the importance of Ser^262^ phosphorylation in AD pathogenesis.

The presence of 14-3-3ζ within the NFTs of AD brains has been reported by a number of studies [68,69]. Various stress-related genes in AD brain were examined, and out of 236 genes analyzed, the expression of 14-3-3ζ was found to be upregulated most significantly [49]. A profound increase in 14-3-3ζ expression was seen in areas affected by NFTs. This study showed that 14-3-3ζ upregulation is an early event and correlates with the severity of AD pathology. Recently, a number of studies have shown that 14-3-3ζ binds to tau and promotes tau phosphorylation and aggregation in vitro [62,81,82,83]. In this study, we showed that overexpression of 14-3-3ζ in rat primary neurons in culture promotes Ser^262^ phosphorylation. Together, these observations suggest that 14-3-3ζ may play a role in the development of AD pathology. 

 Synapses are the structural units of neuronal communication and play an essential role in learning and memory. Synapses are formed, matured, stabilized, remodeled, and eliminated throughout adulthood. Progressive loss of synapses is the major substrate of cognitive decline in patients suffering from AD [30,84]. Synapse loss begins early in the MCI stage and progresses along with the severity of the disease [50]. Biochemical, electron microscopy, and immunohistochemical studies have shown that the synaptic pathology in AD brain originates from the presynaptic terminal and spreads to postsynaptic sites. This is evident by the progressive decline in the level of presynaptic protein synaptophysin levels in the brain [85]. Similar observations have been made in AD mouse models [86]. 

To determine the impact of increased expression of 14-3-3ζ in synapses, we analyzed three synaptic proteins in neurons infected with Ln-14-3-3ζ: presynaptic protein synaptophysin [87], postsynaptic protein PSD-95 [88], and intersynaptic adhesion protein N-Cadherin [89]. We found that 14-3-3ζ overexpression does not affect the levels of PSD-95 or the intracellular levels of N-Cadherin. However, neurons overexpressing 14-3-3ζ displayed significantly reduced synaptophysin levels due to accelerated degradation (Figure 7). A previous study has shown that 14-3-3ζ binds to the cell adhesion molecule L1 and negatively regulates neurite outgrowth in primary neurons in culture [48]. Synaptophysin is a synaptic vesicle protein regulating neurotransmitter release and synaptic plasticity, and is involved in synapse formation [87]. L1, on the other hand, is involved in axonal guidance [90]. Increased expression of 14-3-3ζ in AD brain may, therefore, cause synaptic pathology by inhibiting neurite outgrowth, synapse formation, and synaptic transmission. 

Tau binds to microtubules and promotes microtubule formation *in vitro*. In this study, we found that, compared to Ln-vector infected neurons, the amount of microtubule bound tau is 30% less in Ln-14-3-3ζ infected neurons (Figure 4). This data indicates that overexpression of 14-3-3ζ in neurons inhibits tau binding to microtubules. Phosphorylation at Ser^262^ alone was shown to be sufficient to cause dissociation of tau from microtubules *in vitro* [21], and the level of Ser^262^ phosphorylated tau in 14-3-3ζ overexpressing neurons is 40% more than in those expressing Ln-vector (Figure 3). It is, therefore, likely that in neurons, 14-3-3ζ inhibits tau microtubule binding in part by promoting tau phosphorylation at Ser^262^.

Overexpression of 14-3-3ζ in neurons causes phosphorylation of tau at Ser^262^, destabilizes microtubules, and reduces intracellular synaptophysin levels (Figures 3, 4, 6 and 7). When 14-3-3ζ overexpressing neurons are treated with proteosome inhibitor, tau phosphorylation is not affected, but synaptophysin levels are restored (Figure 7). When 14-3-3ζ overexpressing neurons are treated with the microtubule stabilizing drug taxol, both tau Ser^262^ phosphorylation and synaptophysin degradation are inhibited (Figure 8). Phosphorylation at Ser^262^ destabilizes microtubules [21], and microtubule instability activates the proteosome [76]. It is, therefore, possible that 14-3-3ζ destabilizes microtubules by promoting tau phosphorylation at Ser^262^, and microtubule destabilization then accelerates proteosomal degradation of synaptophysin. 

A recent study has reported that transgenic overexpression of 14-3-3ζ protects the hippocampus against endoplasmic reticulum stress and epileptic seizures in mouse brain [91]. In this study, the authors show that overexpression of 14-3-3ζ in the brain prevents neuronal apoptosis caused by seizure. We have found that when overexpressed in primary neurons in culture, 14-3-3ζ does not promote apoptosis (Figure 2). However, 14-3-3ζ overexpression significantly downregulates synaptophysin protein levels (Figure 6), suggesting its involvement in synapse elimination. It is possible that 14-3-3ζ may exert its anti-apoptotic effects and downregulation of synaptophysin protein activities via independent mechanisms.

Increased expression of various 14-3-3 isoforms is observed in a number of human diseases: 14-3-3σ in colorectal carcinoma [92], 14-3-3β, γ, ζ and τ in lung cancers [93,94], 14-3-3τ in breast cancer [95], 14-3-3β, γ, η (τ), σ and ε in the reactive astrocytes of Creutzfeldt-Jakob disease brain [96], 14-3-3γ in ischemic brain [97,98], 14-3-3τ in amyotrophic lateral sclerosis spinal cord [99], 14-3-3ε in reactive astrocytes of demyelinating lesions of Multiple Sclerosis [100], and 14-3-3ζ in AD brain [49]. The pathological significance of the increased 14-3-3 isoforms in these various disorders is not entirely clear. However, 14-3-3 proteins have been suggested to be oncogenic [101]. Overexpression of 14-3-3τ in breast carcinoma cells promotes proteosomal degradation of Cdk inhibitor p21, which is one of the major regulators of p53-dependent tumor suppression and cellular senescence [95]. In this study, we showed that overexpression of 14-3-3ζ in primary neurons in culture promotes proteosomal degradation of presynaptic protein synaptophysin. It will be interesting to examine if activation of the proteosome is a common mechanism in various human diseases associated with increased levels of different 14-3-3 isoforms.

## Supporting Information

Figure S1 **Disruption of PKA activity inhibits NGF-induced tau Ser^262^ phosphorylation in PC12 cells.** PC12 cells transfected with Myc-PKA-DN or vector were exposed to NGF and then analyzed for tau phosphorylation by Western blot analysis. Based on blot band intensities, relative amount of tau phosphorylation at indicated sites was determined. Values are mean ± S.E. from three determinations. *p< 0.005 with respect to vector transfected and NGF exposed cells.
(TIF)

Figure S2 **Overexpression of 14-3-3ζ does not affect survival of neurons.** Rat hippocamal neurons in culture were infected with Ln-14-3-3ζ or Ln-vector and then analyzed by MTT assay for cell survival (panel A) or by Western blot analysis for active caspase 3. Neurons treated with staurosporine (2 mg/ml) were used as positive controls.
(TIF)

Figure S3 **14-3-3ζ overexpression does not affect synaptophysin transcription in neurons.** Ln-14-3-3ζ or Ln-vector infected rat hippocampal primary neurons in culture were analyzed for the level of synaptophysin mRNA by qRT-PCR.
(TIF)

Figure S4 **Synaptophysin degrdation in 14-3-3ζ overexpressing neurons in not mediated by lysosome.** Ln-vector or Ln-14-3-3ζ infected neurons were treated with indicated lysome inhibitor and analyzed by Western blot analysis.
(TIF)
