# Correction: Overexpression of 14-3-3ζ Promotes Tau Phosphorylation at Ser^262^ and Accelerates Proteosomal Degradation of Synaptophysin in Rat Primary Hippocampal Neurons

**DOI:** 10.1371/journal.pone.0102932

**Published:** 2014-07-10

**Authors:** The PLOS ONE Staff

## Notice of Republication

This article was republished on June 24, 2014, to replace missing Greek characters throughout the text. Please download this article again to view the correct version. The originally published, uncorrected article and the republished, corrected article are provided here for reference.

## Supporting Information

File S1 Originally published, uncorrected article.
(PDF)

File S2 Republished, corrected article.
(PDF)
