# Supplementary figures and images for: Overexpression of 14-3-3ζ Promotes Tau Phosphorylation at Ser262 and Accelerates Proteosomal Degradation of Synaptophysin in Rat Primary Hippocampal Neurons
Source: PLoS One. 2013 Dec 19;8(12):e84615. doi: 10.1371/journal.pone.0084615 (PMC3868614; doi:10.1371/journal.pone.0084615)

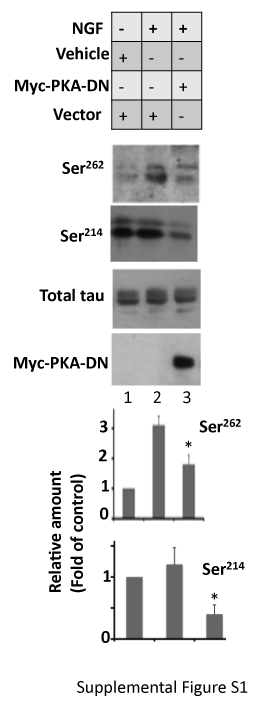

Supplement: Figure S1 — Disruption of PKA activity inhibits NGF-induced tau Ser262 phosphorylation in PC12 cells. PC12 cells transfected with Myc-PKA-DN or vector were exposed to NGF and then analyzed for tau phosphorylation by Western blot analysis. Based on blot band intensities, relative amount of tau phosphorylation at indicated sites was determined. Values are mean ± S.E. from three determinations. *p< 0.005 with respect to vector transfected and NGF exposed cells. (TIF) [file pone.0084615.s001.tif]

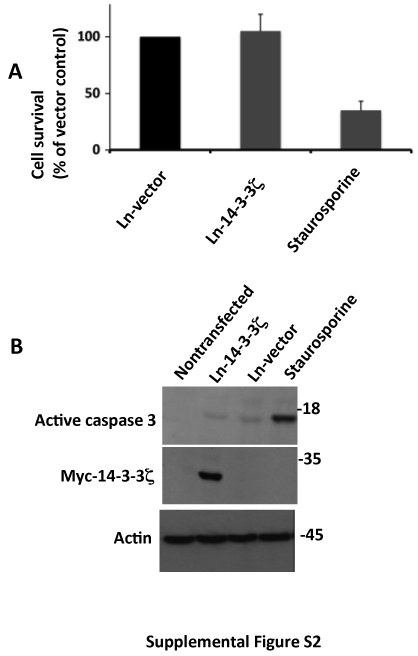

Supplement: Figure S2 — Overexpression of 14-3-3ζ does not affect survival of neurons. Rat hippocamal neurons in culture were infected with Ln-14-3-3ζ or Ln-vector and then analyzed by MTT assay for cell survival (panel A) or by Western blot analysis for active caspase 3. Neurons treated with staurosporine (2 mg/ml) were used as positive controls. (TIF) [file pone.0084615.s002.tif]

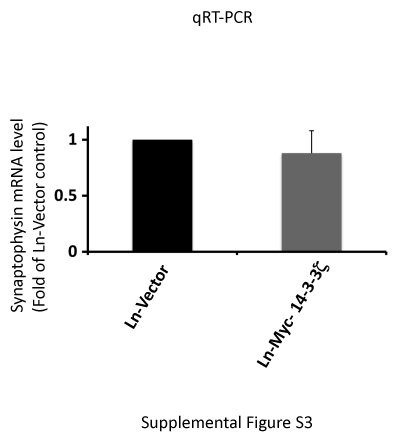

Supplement: Figure S3 — 14-3-3ζ overexpression does not affect synaptophysin transcription in neurons. Ln-14-3-3ζ or Ln-vector infected rat hippocampal primary neurons in culture were analyzed for the level of synaptophysin mRNA by qRT-PCR. (TIF) [file pone.0084615.s003.tif]

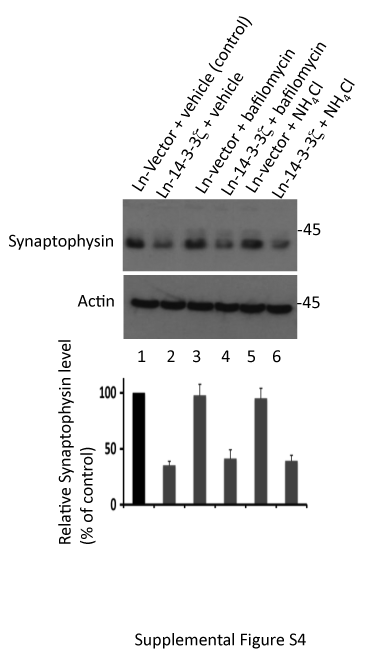

Supplement: Figure S4 — Synaptophysin degrdation in 14-3-3ζ overexpressing neurons in not mediated by lysosome. Ln-vector or Ln-14-3-3ζ infected neurons were treated with indicated lysome inhibitor and analyzed by Western blot analysis. (TIF) [file pone.0084615.s004.tif]
