# Supplementary material for: Correction: Overexpression of 14-3-3ζ Promotes Tau Phosphorylation at Ser262 and Accelerates Proteosomal Degradation of Synaptophysin in Rat Primary Hippocampal Neurons
Source: PLoS One. 2014 Jul 10;9(7):e102932. doi: 10.1371/journal.pone.0102932 (PMC4092143; doi:10.1371/journal.pone.0102932)
Supplement: File S2 — Republished, corrected article. (PDF) [file pone.0102932.s002.pdf]

Overexpression of 14-3-3ζ Promotes Tau Phosphorylation at Ser^{262} and Accelerates Proteosomal Degradation of Synaptophysin in Rat Primary Hippocampal Neurons

Hamid Y. Qureshi[1☯], Dong Han[1☯], Ryen MacDonald[1,2], Hemant K. Paudel[1,2*]

1 The Bloomfield Center for Research in Aging, Lady Davis Institute for Medical Research, Jewish General Hospital, Montreal, Quebec, Canada, **2** Department of Neurology and Neurosurgery, McGill University, Montreal, Quebec, Canada

Abstract

β-amyloid peptide accumulation, tau hyperphosphorylation, and synapse loss are characteristic neuropathological symptoms of Alzheimer's disease (AD). Tau hyperphosphorylation is suggested to inhibit the association of tau with microtubules, making microtubules unstable and causing neurodegeneration. The mechanism of tau phosphorylation in AD brain, therefore, is of considerable significance. Although PHF-tau is phosphorylated at over 40 Ser/Thr sites, Ser^{262} phosphorylation was shown to mediate β-amyloid neurotoxicity and formation of toxic tau lesions in the brain. *In vitro*, PKA is one of the kinases that phosphorylates tau at Ser^{262}, but the mechanism by which it phosphorylates tau in AD brain is not very clear. 14-3-3ζ is associated with neurofibrillary tangles and is upregulated in AD brain. In this study, we show that 14-3-3ζ promotes tau phosphorylation at Ser^{262} by PKA in differentiating neurons. When overexpressed in rat hippocampal primary neurons, 14-3-3ζ causes an increase in Ser^{262} phosphorylation, a decrease in the amount of microtubule-bound tau, a reduction in the amount of polymerized microtubules, as well as microtubule instability. More importantly, the level of pre-synaptic protein synaptophysin was significantly reduced. Downregulation of synaptophysin in 14-3-3ζ overexpressing neurons was mitigated by inhibiting the proteosome, indicating that 14-3-3ζ promotes proteosomal degradation of synaptophysin. When 14-3-3ζ overexpressing neurons were treated with the microtubule stabilizing drug taxol, tau Ser^{262} phosphorylation decreased and synaptophysin level was restored. Our data demonstrate that overexpression of 14-3-3ζ accelerates proteosomal turnover of synaptophysin by promoting the destabilization of microtubules. Synaptophysin is involved in synapse formation and neurotransmitter release. Our results suggest that 14-3-3ζ may cause synaptic pathology by reducing synaptophysin levels in the brains of patients suffering from AD.

Citation: Qureshi HY, Han D, MacDonald R, Paudel HK (2013) Overexpression of 14-3-3ζ Promotes Tau Phosphorylation at Ser^{262} and Accelerates Proteosomal Degradation of Synaptophysin in Rat Primary Hippocampal Neurons. PLoS ONE 8(12): e84615. doi:10.1371/journal.pone.0084615

Editor: Efthimios M. C. Skoulakis, Alexander Fleming Biomedical Sciences Research Center, Greece

Received June 21, 2013; **Accepted** November 15, 2013; **Published** December 19, 2013

Copyright: © 2013 Qureshi et al. This is an open-access article distributed under the terms of the Creative Commons Attribution License, which permits unrestricted use, distribution, and reproduction in any medium, provided the original author and source are credited.

Funding: This work was supported by grants from Canadian Institute for Health Research, and Alzheimer's Society of Canada. The funders had no role in study design, data collection and analysis, decision to publish, or preparation of the manuscript.

Competing interests: The authors have declared that no competing interests exist.

* E-mail: hemant.paudel@mcgill.ca

☯ These authors contributed equally to this work.

Introduction

Senile plaques and neurofibrillary tangles (NFTs) are the characteristic neuropathological hallmarks found in the brains of patients suffering from Alzheimer's disease (AD). Plaques are made up of β-amyloid peptides derived from amyloid precursor protein cleavage, and NFTs mainly contain paired helical filaments (PHFs), which are composed of hyperphosphorylated, fibrillar, microtubule-associated protein tau [1,2]. Hyperphosphorylated, fibrillar tau is also found in numerous neurodegenerative diseases that are collectively known as tauopathies, which include Picks disease, progressive supranuclear palsy, corticobasal degeneration and frontotemporal dementia (FTDP-17) [1]. Mutations in genes encoding for tau have been observed in the familial type of FTDP-17. These mutations result in tau hyperphosphorylation and fibrillization in experimental models both *in vivo* and *in vitro* [3,4,5,6,7,8,9,10,11]. In these tauopathies, neurodegeneration occurs in the absence of β-amyloid pathology [12]. Furthermore, studies using transgenic mice, primary neurons, and drosophila have shown that tau is required for β-amyloid neurotoxicity [13,14,15,16]. Tau dysfunction has been recognized as a central pathology in the development of AD.

Tau is a neuron-specific microtubule-associated protein. In normal brain, it binds to and promotes the formation and stability of microtubules [2]. However, PHF-tau (tau isolated from PHFs) is hyperphosphorylated and does not bind to microtubules. Upon dephosphorylation, PHF-tau regains its

microtubule-binding ability, suggesting that hyperphosphorylation prevents tau from associating with microtubules, leading to microtubule instability and eventual neurodegeneration in AD brain [17]. PHF-tau is phosphorylated at over 40 Ser/Thr sites [18,19,20]. In addition, it has also been reported that Tyr^{18}, Tyr^{197} and Tyr^{394} are phosphorylated in PHFs [2]. Among these sites, Ser^{256}, Ser^{262}, Ser^{289} and Ser^{356} are located within the microtubule-binding region of tau [20]. The impact of phosphorylation at Ser^{262} has been studied the most, and phosphorylation at this site alone significantly reduces the affinity of tau for microtubules, and is sufficient in causing microtubule instability *in vitro* and *in vivo* [21].

In addition, in both primary neurons and drosophila, Ser^{262} tau phosphorylation mediates β-amyloid peptide toxicity in the brain [15,16]. Cdk5 and GSK3β are considered two of the main kinases that phosphorylate tau in AD brain [22,23,24,25,26,27,28]. In the drosophila model of tauopathy, tau Ser^{262} phosphorylation is a prerequisite for tau phosphorylation by Cdk5 and GSK3β [29]. Finally, synapse loss is regarded as the basis for dementia in AD patients [30]. Phosphorylation of tau at Ser^{262} causes a loss of pre- and postsynaptic proteins and reduces the number of dendritic spines in neurons [16]. These studies suggest that phosphorylation of tau at Ser^{262} plays an important role in the development of AD. *In vitro*, Ser^{262} is phosphorylated by a number of kinases including MARK, PKA, PKC, CamKII, and phosphorylase kinase [31,32,33,34]. How these kinases phosphorylate tau at Ser^{262} *in vivo* and in AD brain is not clearly understood.

Understanding tau phosphorylation in the normal brain may provide insight into the mechanisms of abnormal tau phosphorylation in AD. Tau phosphorylation is developmentally regulated. In fetal brain cells that are still dividing, tau is highly phosphorylated [35]. As these cells differentiate into neurons and the brain develops into an adult state, tau phosphorylation, at many sites, becomes undetectable [35]. Interestingly, several of the same tau sites that are phosphorylated in AD are also phosphorylated in a normal, fetal brain during development [35,36,37,38,39]. It has been suggested that in the developing brain, tau phosphorylation is regulated by cell cycle mechanisms that reappear in AD brain [19,35,36,37,38,39]. Examining tau phosphorylation during brain development, therefore, may assist in determining the mechanism of tau hyperphosphorylation in AD. Neural development encompasses the differentiation of neurons from precursor cells. When dividing PC12 cells are exposed to Nerve Growth Factor (NGF), they slowly stop proliferating, develop neurites and growth cones, and differentiate into neurons. NGF-exposed PC12 cells are widely used to study neuronal differentiation [40,41,42,43,44].

To study how tau is phosphorylated during the transition of mitotic cells to terminally differentiated neurons, we have used NGF-exposed PC12 cells and found that adaptor protein 14-3-3ζ promotes tau phosphorylation at Ser^{262} by PKA in differentiating neurons. 14-3-3 are a family of proteins that regulate many different cellular functions including cell cycle, apoptosis, and signal transduction [45,46]. There are seven 14-3-3 isoforms in mammalian cells (β, ε, γ, η, σ, θ and ζ). Among these isoforms, 14-3-3ζ is involved in brain development and neuronal differentiation [47,48], and is upregulated in AD brain [49]. Therefore, to evaluate the pathological significance of our findings, we overexpressed 14-3-3ζ in rat primary neurons in culture. Herein, we report that overexpression of 14-3-3ζ promotes tau phosphorylation at Ser^{262} and causes proteosomal degradation of the presynaptic protein synaptophysin in primary neurons in culture. Loss of synaptophysin is recognized as an indicator of synaptic pathology in AD brain [30,50]. Our study suggests that 14-3-3ζ causes synaptic loss by destabilizing microtubules, leading to proteosomal degradation of synaptophysin in the neurons of patients suffering from AD.

Materials and Methods

Chemicals, Proteins and Antibodies

Olomoucine, forskolin, PKA inhibitor P9115 (the cell permeable myristoylated PKI inhibitor peptide 14-22), and PKAc (the catalytic subunit of PKA) were purchased from Sigma. Recombinant longest human tau isoform was purified from bacterial lysate [11]. Tau phosphorylation-specific antibodies used for the study were PHF-1 (1:1000; for phosphorylated $Ser^{396/404}$), AT8 (1:500; for phosphorylated $Ser^{202/205}$), 12E8 (1:500; for phosphorylated Ser^{262}), pS214 (1:1000; for phosphorylated Ser^{214}) and pS262 (1:1000; for phosphorylated Ser^{262}), and were described previously [51]. AT180 (1:1000; for Thr^{231}phosphorylated tau) was from Innogenetics. Monoclonal antibodies Tau 5 (1:1000; for total tau), anti-14-3-3ζ (1:500), anti-Myc (1:1000) and anti-Flag (1:1000) are also described previously [11,52]. Monoclonal antibody against synaptophysin (1:1000) was from Sigma. Anti-PSD-95 (1:500) and anti-N-cadherin (1:500) monoclonal antibodies were from Cell Signaling Technology Inc. Monoclonal anti-Ac-tubulin (1:250) and anti-Tyr-tubulin (1:250) were from Sigma. A polyclonal anti-synaptophysin (1:1000), and a monoclonal anti-active caspase 3 (1:200) were purchased from Millipore. Protein concentrations of each cell lysate were determined by the BioRad protein assay using BSA as the standard. Equal amounts of protein from each sample were separated on SDS-PAGE and then Western blotted. Primary antibody incubation (overnight) was followed by incubation with either rabbit polyclonal or mouse monoclonal secondary antibody linked to horseradish peroxidase (1:2000) for 4 hr. Immunoreactivity was visualized by using enhanced chemiluminescence reagent and exposure to X-ray film. Films were scanned densitometrically, and the optical density of bands was quantified using Image J software (NIH).

Lentivirus Production, Neuronal Culture and Viral Infection

Human Myc-14-3-3ζ gene was subcloned into the pcDNA3 at Age1/Not1 sites using forward (5'- AAAAACCGGTATG GATAAAAATGAGCTG-3') and reverse (5'-AAAAGCGGCCGCCTACAGATCTTCTTCAGAAATAAGTTTTT GTTCATTTTCCCCTCCTTCTCC-3') primers. Myc-14-3-3ζ was subsequently subcloned into lentiviral vector pTet07 CSII-CMV-mcs-ires-GFPq, as described [51]. The viral DNA was

transfected into 293SF-PacLV29-6 packaging cells. Virus was collected from the cell supernatant, concentrated, and its titer was determined. Hippocampal neurons were cultured from the E18 rat pups [51]. Neurons in culture for two weeks were infected with lentivirus (MOI of 10). After 48 hr of infection, neurons were lysed in lysis buffer (50 mM Tris-HCl (pH 7.4), 0.1 mM EDTA, 0.1 mM DTT, 150 mM NaCl, 50 mM β-glycerol phosphate, 0.1 mM EGTA, 10 mM $MgCl_2$, 0.2% Nonidet P-40) and supplemented with phosphatase and protease inhibitor cocktail (Sigma), and subsequently analyzed.

cDNA Cloning, Cell Culture and Transfection

Murine PKA catalytic subunit (PKAc) and its dominant negative mutant PKA-DN (K272H) both in the $pRSET_B$ vector (gifts of Dr. Susan Taylor of University of California, San Diego) were subcloned with a Myc tag into pcDNA3.1 by PCR at BamH1/ Nde1 site using forward 5'-ggt acc at ATG GGC AAC GCC GCC GCC GCC-3 and reverse 5' - GGT ACC AAA CTC AGT AAA CTC CTT GCC -3' primers. Dominant negative 14-3-3ζ (K49E) was a gift from Dr. Haian Fu of the Emory University, Atlanta, Georgia. PC12 cells were exposed to NGF (2.5 S) (0.1μg/ml) and their morphology was observed under the Nikon microscope as described [40,41]. In some experiments, PC12 cells were first transfected with the indicated cDNA for 6 hr using Lipofectamine 2000 and then NGF was added [41].

Drug Treatments

PC12 cells exposed with NGF for six days were treated with P9115 (0.5 μM), EGTA (2 mM), LiCl (10 mM) or olomoucine (60 μM) for 1 hr, and harvested as described previously [41]. To inhibit lysosome, rat primary hippocampal neurons were infected with Ln-14-3-3ζ and were treated with bafilomycin (10 nM) or NH_4Cl (10 mM) for 24 hr. To inhibit protein synthesis, infected neurons were treated with cycloheximide (10 μg/ml) for 2, 4, 8, 16 and 24 hr. To inhibit the proteosome, infected neurons were treated with MG132 (15 μM) or lactacystin (2.5 μM) for 24 hr as described [53].

Immunocytochemistry

Infected neurons were fixed with 4% paraformaldehyde in PBS at room temperature for 30 min. Fixed neurons were washed and permeabilized by incubating with PBS containing 0.1% Triton X-100 and 1% BSA for 30 min at room temperature. Neurons were then incubated for 12 hr at 4 °C with anti-synaptophysin mouse monoclonal (Sigma), 1:200 or rabbit polyclonal anti-Myc (Sigma-Aldrich), 1:200; washed and then labeled by incubating with Alexa Fluro 488 or Cy3 conjugated goat anti-mouse or goat anti-rabbit for 1 h at room temperature.

Image Acquisition and Quantification

Images were obtained using Leica AF 6500 immunofluorescent microscope (Germany). Labeled transfected neurons were chosen randomly for quantification from 3-4 coverslips. The number of immunostained pucta were counted using MetaMorph Image analysis software (Universal Imaging Corporation).

In vitro Kinase Assay

PKA and Cdk5 activities were assayed as described previously using Kemptide (LRRASLG) and KTPKKAKKPKTPKKAKKI peptides, respectively [34]. In an assay mixture containing 50 mM Tris-HCl (pH 7.4), 1 mM DTT, 0.1 mM EDTA, 10 mM $MgCl_2$, 0.5 mM [γ^{32}P]ATP, the respective substrate peptides (20 μM), and phosphatase and protease inhibitor cocktails, an aliquot of cell lysate was added to initiate the reaction. After 30 min at 30 °C, the reaction was stopped by the addition of an equal volume of 20% TCA to each sample. Samples were placed on ice for 20 min and then centrifuged using a bench top centrifuge. The supernatant of each sample (20 μl) was analyzed using a filter paper assay to determine the amount of ^{32}P incorporated into the peptide substrate [54].

Quantitative Real Time PCR

Total RNA was isolated from neurons infected with Ln-14-3-3ζ or Ln-vector using an RNeasy mini kit (Qiagen). Total RNA (1 μg) was used for the first-strand synthesis with reverse transcriptase II using oligodT or random primer (Invitrogen). SYBR® Green based (Qiagen) real-time PCR was performed with gene specific primers for synaptophysin (Rn-Syp-2-SG) and GAPDH (Rn-GAPDH-1-SG) (Qiagen). Data was analyzed by Real-Time PCR software 7500 version 2.0.4 (Applied Biosystems). The relative RNA expression of the genes of interest were determined using the comparative ΔΔCt method with GAPDH as the endogenous control.

Cell Survival

MTT cell survival assay was carried out as described previously, using a kit from ATCC and following the manufacturer's instruction manual [53]. Primary neurons in a 96-well plate were infected with Ln-14-3-3ζ or Ln-vector and incubated with MTT reagent at 37 °C in the dark. After 2 hr of incubation, the reaction was stopped and absorbance at 570 nm was monitored using an ELISA plate reader to quantify cell viability [53].

Microtubule Sedimentation Assay

A microtubule sedimentation assay was performed as described previously [55]. Neurons were lysed in Pipes buffer (0.1 M PIPES (pH 6.6), 1 mM EGTA, 1 mM $MgSO_4$, 1 mM β-mercaptoethanol) containing 0.2% Nonidet P-40 as well as protease and phosphatase inhibitor cocktail. Each lysate was adjusted to 1 mg/ml protein concentration on ice and was transferred to a water bath at 37 °C. After 5 min, pre-warmed GTP (1 mM) and taxol (10 μM) were added to each sample. After 30 min of incubation at 37° C, samples were centrifuged at room temperature. The supernatant was designated as S. The pellet was gently washed with warm Pipes buffer containing GTP and taxol and was designated as P. Both S and P were analyzed by Western blot analysis.

Statistics

The data is expressed as the mean ± SEM and was analyzed by one-way or two-way ANOVA followed by Bonferroni's *post hoc* test for multigroup and the student's t-test for two group comparisons. Differences with $p<0.05$ were considered significant.

Results

Phosphorylation of Tau in NGF-Exposed PC12 Cells

PC12 cells did not display any obvious signs of differentiation during the first day of NGF exposure. After 1 day (24 hr) of NGF exposure, a few cells had developed protrusions. After 2 days (48 hr), ~50% cells had neurites of various lengths. By day 6, >90% cells were fully differentiated, displaying long neurites and growth cones (data not shown).

To determine tau phosphorylation during PC12 cell differentiation, we probed NGF-exposed PC12 cell extracts with different antibodies that recognize tau phosphorylated site-specifically. As shown in Figure 1A, total tau level increased with NGF exposure time until day one (lanes 1-4), and then remained constant thereafter (lanes 4-6). Basal level of tau phosphorylation was observed in untreated cells (lane 1) and, upon NGF exposure, phosphorylation at all sites appeared to increase (lanes 2-6). However, when tau phosphorylation at each site was normalized against total tau, phosphorylation at proline-directed sites $Ser^{396/404}$ and $Ser^{202/205}$ were found to increase several fold within minutes of NGF exposure, but phosphorylation at Thr^{231} remained unchanged (Figure 1A, lanes 2-3). After one day of exposure, phosphorylation at both $Ser^{396/404}$ and $Ser^{202/205}$ sites decreased compared to the cells exposed for 60 min, but remained higher than the basal level (Figure 1A, lane 4 and B). After 2 and 6 days of NGF exposure, phosphorylation at both $Ser^{396/404}$ and $Ser^{202/205}$ sites remained comparable to that of 1 day of exposure. Phosphorylation at Thr^{231}, however, remained unchanged throughout (Figure 1B).

Tau was phosphorylated in naive PC12 cells at the non-proline-directed sites Ser^{214} and Ser^{262} (Figure 1A, lane 1). Phosphorylation at Ser^{214} remained at basal level throughout the exposure to NGF (Figure 1A, lanes 2-6 and B). Ser^{262} phosphorylation (probed by 12E8 antibody), on the other hand, slowly increased during the initial phase (lanes 2-3), but surged to 2.2, 2.1 and 2.0 fold higher than the basal level on days 1, 2 and 6, respectively (Figure 1B). Similar observations were made when 12E8 antibody was replaced by pS262 antibody (data not shown). Thus, Ser^{262} phosphorylation became prominent at day 1 and remained at this level thereafter. As shown in Figure 1B, proline-directed $Ser^{396/404}$ and $Ser^{202/205}$ phosphorylation and non-proline-directed Ser^{262} phosphorylation in NGF exposed PC12 cells occurred sequentially. Proline-directed $Ser^{396/404}$ and $Ser^{202/205}$ phosphorylation occurred first, and as they began to decline, the non-proline-directed Ser^{262} phosphorylation began to increase, which peaked and was sustained at peak level.

Identification of Kinases

GSK3β is one of the major kinases that phosphorylates tau in the brain [25,26,27,56]. However, when NGF-exposed cells

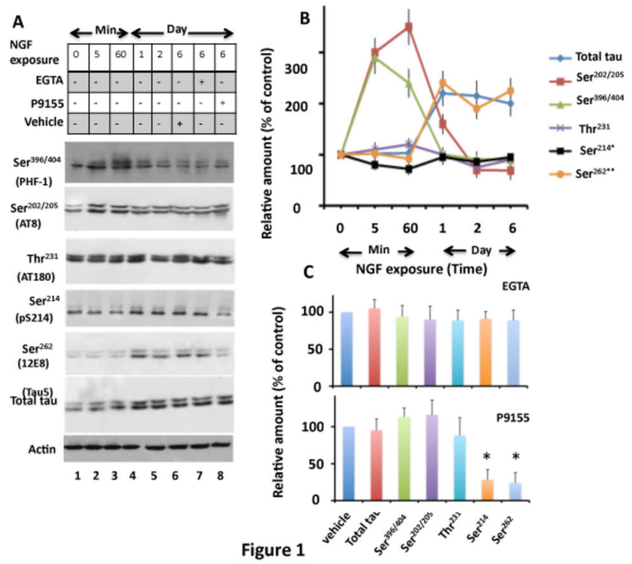

Figure 1. Phosphorylation of tau in NGF-exposed PC12 cells. PC12 cells exposed to NGF for the indicated time points followed by EGTA or P9115 were analyzed for tau phosphorylation by Western blot analysis. (A) Western blots. (B) Relative amount. The relative amount of phosphorylated tau at each site at the indicated time points was determined by normalizing the band intensity of phosphorylated tau with the respective tau band. The relative amount of total tau was determined by normalizing the tau band to the respective actin band. (C) Effects of EGTA and P9115. Band intensity values of tau or phosphorylated tau at indicated sites in cells exposed to NGF for 6 days and treated with EGTA (panel A, lane 7) or P9115 (panel A, lane 8) were normalized as in panel B and are expressed as the % of vehicle-treated control (panel A, lane 6). Values in panels B and C with standard error are the average of three determinations from three cultures. $*p < 0.005$ with respect to vehicle-treated cells.

doi: 10.1371/journal.pone.0084615.g001

were treated with the GSK3β inhibitor LiCl (10 mM), tau phosphorylation at all of the examined sites including $Ser^{396/404}$, $Ser^{202/205}$, Thr^{231}, Ser^{214} and Ser^{262} was similar when compared to the vehicle treated control cells (data not shown). This data is consistent with previous reports [57] and indicates that NGF does not activate GSK3β in PC12 cells. When LiCl was replaced by the Cdk5 inhibitor olmoucine [41], phosphorylation at both $Ser^{396/404}$ and $Ser^{202/205}$ was reduced by 78.9 and 69.5%, respectively, when compared to vehicle-treated controls (data not shown). This result is as expected and indicates that Cdk5 phosphorylates tau at both $Ser^{396/404}$ and $Ser^{202/205}$ sites in differentiated PC12 cells [51]. To identify the kinases responsible for non-proline-directed phosphorylation, we treated NGF-exposed PC12 cells with EGTA, a specific chelator of Ca^{2+}, and analyzed them for tau protein phosphorylation (Figure 1A, lane 7). Phosphorylation at $Ser^{396/404}$, $Ser^{202/205}$, and Thr^{231} was similar in EGTA and vehicle treated cells (Figure 1C). Likewise, phosphorylation at Ser^{214} and Ser^{262} was also unaltered by EGTA (compare lanes 6 and

7 in Figure 1A and see Figure 1C)). Thus, depletion of Ca^{2+} did not inhibit tau phosphorylation at all of the examined sites. This result determined that Ca^{2+}-dependent kinases PKC, CamKII, and phosphorylase kinase do not phosphorylate tau in NGF-exposed PC12 cells.

PKA phosphorylates tau *in vitro* and *in vivo* [33,58,59,60]. To determine if PKA was involved, we treated NGF-exposed PC12 cells with the cell permeable PKA inhibitor P9115. P9115 did not affect tau phosphorylation at $Ser^{396/404}$, $Ser^{202/205}$ or Thr^{231}, but inhibited phosphorylation at Ser^{214} (Figures 1A, lane 8 and 1C), as expected [59]. Interestingly, P9115 also inhibited phosphorylation at Ser^{262} by ~76% (Figure 1C). To substantiate this result, we transfected PC12 cells with a dominant negative PKA mutant, Myc-PKA-DN, and exposed them to NGF (Figure S1). Tau phosphorylation at Ser^{262} and Ser^{214} were 42.1% and 65%, respectively less in Myc-PKA-DN transfected cells when compared to respective vector transfected controls (compare lane 2 with lane 3). Based on this result, we concluded that PKA phosphorylates tau at Ser^{214} and Ser^{262} in NGF-exposed PC12 cells.

NGF Does Not Activate PKA in PC12 Cells

PKA exists as an inactive dimer, composed of a catalytic PKAc subunit and an inhibitory subunit. Dissociation of the inhibitory subunit from the holoenzyme via cAMP signaling activates PKAc [61]. To test if NGF activates PKA, we measured PKA activity by an *in vitro* kinase assay in PC12 cell extract exposed to NGF or the PKA agonist forskolin. Forskolin activated PKA but not Cdk5, as expected (Figure 2A, lower panel). However, NGF activated Cdk5 but had no effect on PKA activity (Figure 2A, upper panel). This result determined that NGF does not activate PKA in PC12 cells.

14-3-3ζ is Involved in Tau Phosphorylation in Mammalian Cells

Previous studies have reported that, *in vitro*, PKA phosphorylates tau at Ser^{262} [31,33] and that, 14-3-3ζ binds to tau and promotes Ser^{262} phosphorylation by PKA [62]. Although a rise in intracellular cAMP level activates PKA *in vivo*, a basal PKA activity has been observed in a number of cell lines including PC12 cells [63,64,65]. Furthermore, when PC12 cells are exposed to NGF, 14-3-3ζ expression significantly increases [66]. NGF, therefore, may promote tau phosphorylation via basal PKA activity at Ser^{262} indirectly by enhancing 14-3-3ζ level in cells. To test this idea, we transfected PC12 cells with a dominant negative 14-3-3ζ mutant Myc-14-3-3ζ (K49N) and then exposed them to NGF. Myc-14-3-3ζ (K49N) inhibited NGF-induced Ser^{262} phosphorylation by 40% but did not affect phosphorylation at Ser^{214}, $Ser^{396/404}$ or $Ser^{202/205}$ (Figure 2B, lane 2). To substantiate this result, we transfected HEK-293 cells with Flag-tau, Myc-PKAc, and different amounts of Myc-14-3-3ζ, and analyzed tau phosphorylation at Ser^{262} and Ser^{214}. PKA phosphorylated tau at both Ser^{214} and Ser^{262}. However, Myc-14-3-3ζ promoted tau phosphorylation at Ser^{262} but not at Ser^{214} (Figure 2C, lanes 4-6). Similar observations were made when pS262 antibody was used (data not shown). Based on these data, we concluded that 14-3-3ζ promotes tau Ser^{262} phosphorylation in intact cells.

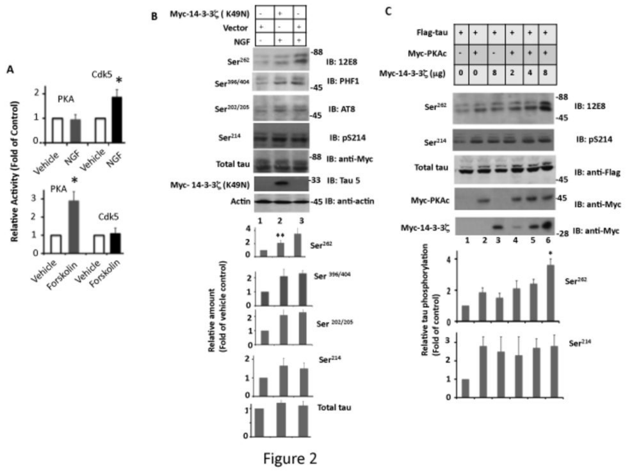

Figure 2. 14-3-3ζ promotes PKA-catalyzed Ser^{262} tau phosphorylation in PC12 and HEK-293 cells. (A) NGF does not activate PKA in PC12 cells. PC12 cells were treated with forskolin, NGF, or vehicle for 24 hr. Treated cells were lysed and each lysate was assayed for PKA or Cdk5 activity using the respective peptide substrate. Values are the average of three determinations from three cultures, and are expressed as the fold change from vehicle-treated control cells. *$p<0.005$ with respect to vehicle treated cells. (B) Disruption of 14-3-3ζ function inhibits tau phosphorylation at Ser^{262} in NGF-exposed PC12 cells. PC12 cells transfected with Myc-14-3-3ζ (K49N) or empty vector were exposed to NGF for 24 hr and then analyzed by Western blotting as in Figure 1. Values with standard error are the average of three determinations from three cultures. **$p < 0.001$ with respect to vector transfected and NGF-treated cells. (C) 14-3-3ζ promotes PKA-catalyzed tau Ser^{262} phosphorylation in HEK293 cells. HEK293 cells co-transfected with Flag-tau and Myc-14-3-3ζ were analyzed by Western blotting. The relative amount of Ser^{262} phosphorylated tau was determined by normalizing the Ser^{262} band in each lane to the respective Flag-tau band. Values with standard error are the average of three determinations from three cultures. *$p < 0.005$ with respect to cells transfected with Flag-tau and Myc-PKAc.

doi: 10.1371/journal.pone.0084615.g002

Overexpression of 14-3-3ζ Promotes Tau Phosphorylation at Ser^{262} and Inhibits Tau Microtubule Binding in Rat Primary Neurons in Culture

Ser^{262} is phosphorylated in PHF-tau [18,67], and phosphorylation at this site causes microtubule instability and neurodegeneration in models both *in vitro* and *in vivo* [15,16,21,29,55]. 14-3-3ζ, on the other hand, was reported to be upregulated in AD brain and localized in neurons with high NFT densities [49,68,69]. We found that 14-3-3ζ promotes tau Ser^{262} phosphorylation during neuronal differentiation (Figure 1). To evaluate the pathophysiological significance of these observations, we infected rat primary hippocampal neurons with lentivirus expressing human Myc-14-3-3ζ (Ln-14-3-3ζ) and analyzed them for tau phosphorylation.

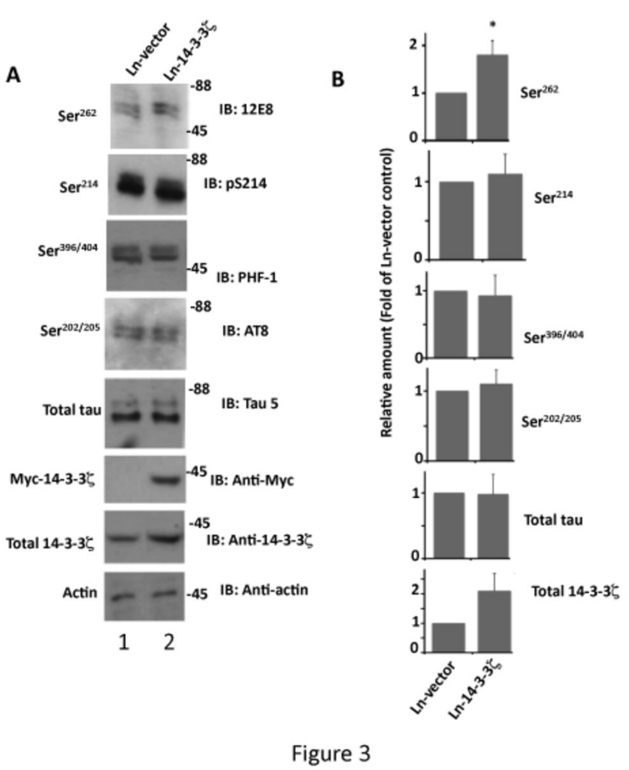

Figure 3. Overexpression of 14-3-3ζ promotes tau phosphorylation at Ser^{262} in rat hippocampal primary neurons in culture. Rat hippocampal primary neurons in culture infected with Ln-14-3-3ζ or Ln-vector were analyzed for tau phosphorylation by Western blotting as in Figure 1. (A) Western blots, (B) Relative amounts. Values with standard error are the average of three determinations from three cultures. *$p < 0.001$ with respect to Ln-vector infected neurons.

doi: 10.1371/journal.pone.0084615.g003

14-3-3ζ overexpression did not change tau levels and did not affect tau phosphorylation at Ser$^{396/404}$, Ser$^{202/205}$ or Ser^{214} significantly, but caused increased 12E8 immunoreactivity by ~1.8-fold (Figure 3A, B) and pS262 immunoreactivity by 2.1-fold (data not shown). Thus, as observed in HEK-293 cells, overexpression of 14-3-3ζ promotes tau phosphorylation at Ser^{262} in neurons.

In neurons, tau binds to microtubules and promotes microtubule polymerization. Ser^{262} phosphorylation significantly affects the tau-microtubule interaction *in vitro* [21]. Since 14-3-3ζ promotes tau phosphorylation at this site, it is possible that 14-3-3ζ overexpression may affect tau microtubule binding. To examine this possibility, we performed a microtubule sedimentation assay using neurons infected with Ln-14-3-3ζ and Ln-vector.

As shown in Figure 4A, in Ln-vector infected neurons, 70.7% of the total tubulin formed microtubules and settled in the pellet (P) (lane 2). In 14-3-3ζ infected neurons, on the other hand, 50.2% of the total tubulin formed microtubules (lane 4). Thus, compared to Ln-vector infected neurons, Ln-14-3-3ζ infected neurons had a 29.1% reduction in the amount of polymerized microtubules (Figure 4B). In Ln-vector infected control neurons, 65% of the total tau bound to microtubules and settled in the microtubule pellet (Figure 4B, lane 2). In Ln-14-3-3ζ infected neurons, however, 45% of the total tau was microtubule bound (lane 4), a reduction of 30.7% compared to Ln-vector infected neurons (Figure 4B).

The relative amount of Ser^{262} phosphorylated tau was 2.8-fold more in Ln-14-3-3ζ infected neurons than in Ln-vector infected (Figure 4B). However, in all neurons Ser^{262} phosphorylated tau was exclusively in the supernatant (Figure 4A, lanes 1 and 3) and was undetectable in the microtubule pellet (Figure 4A, lanes 2 and 4). This result indicates that Ser^{262} phosphorylated tau does not bind to microtubules. Thus, 14-3-3ζ overexpression promotes tau phosphorylation at Ser^{262}, inhibits tau microtubule binding, and reduces the amount of polymerized microtubules in the neurons.

14-3-3ζ Overexpression Destabilizes Microtubules in Rat Primary Neurons in Culture

Tau binds to and promotes the stability of microtubules *in vitro* [2,11]. Since 14-3-3ζ overexpression inhibited tau microtubule binding (Figure 4), we examined microtubule stability in neurons infected with Ln-14-3-3ζ. 14-3-3ζ overexpression did not affect tubulin turnover, as the level of tubulin was similar in 14-3-3ζ and vector overexpressing neurons (Figure 5A). However, compared to Ln-vector infected neurons, the relative amount of stable Ac-tubulin was 42% less in 14-3-3ζ expressing neurons (Figure 5A). Likewise, the relative amount of Tyr-tubulin representing unstable microtubules was 30% more in 14-3-3ζ overexpressing neurons than in those expressing vector (Figure 4A). Under the fluorescent microscope, Ln-vector infected neurons had a relatively weak Tyr tubulin signal in the cell body and in neurites. In Ln-14-3-3ζ infected neurons, on the other hand, there was an intense Tyr-tubulin signal in the cell body and in hillocks (Figure 5B, lower). Total tubulin immunostaining was similar in both Ln-14-3-3ζ and Ln-vector infected neurons. These data determined that 14-3-3ζ overexpression destabilizes the microtubule cytoskeleton in rat hippocampal primary neurons in culture.

Overexpression of 14-3-3ζ Downregulates Synaptophysin Protein Level in Neurons

Previous studies have shown that tau phosphorylated at Ser^{262} promotes neurodegeneration [29]. 14-3-3ζ enhances Ser^{262} phosphorylation in neurons (Figure 3). Therefore, to evaluate the pathological impact of 14-3-3ζ overexpression, we first performed an MTT cell survival assay of neurons infected with either Ln-vector or Ln-14-3-3ζ. The % of live cells in 14-3-3ζ expressing neurons was similar (105.1 ± 15) to those expressing vector control (Figure S2A). When Western blotted for active caspase 3, the respective bands resulting from 14-3-3ζ and vector expressing neuronal lysates were barely detectable (Figure S2B). Thus, overexpression of 14-3-3ζ did not affect cell survival and did not promote apoptosis in neurons.

14-3-3ζ is present in synapses [46], and synapse loss precedes neurodegeneration in AD [70]. Primary neurons in

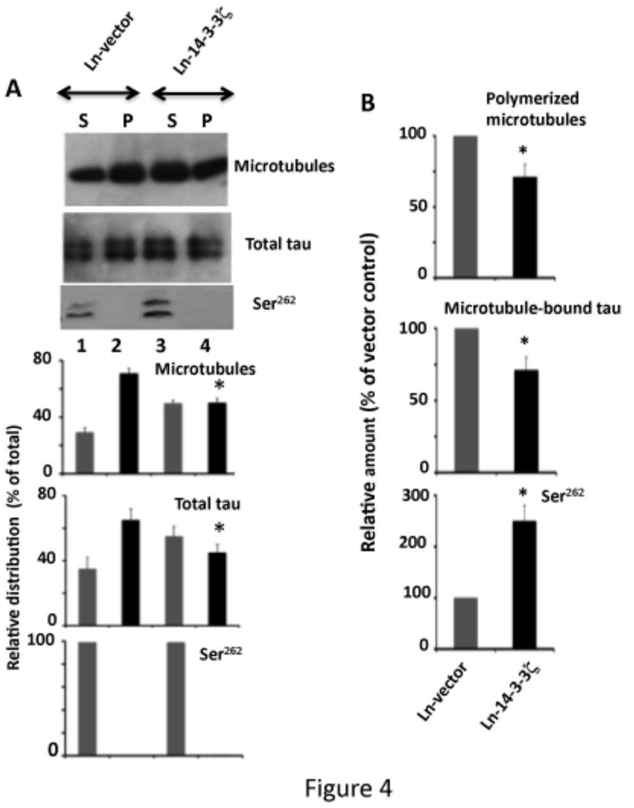

Figure 4. Overexpression of 14-3-3ζ inhibits tau binding to microtubules and reduces the amount of polymerized microtubules in rat hippocampal primary neurons in culture. Primary neurons infected with Ln-14-3-3ζ or Ln-vector were subjected to a microtubule sedimentation assay. The resulting microtubule pellet (P) and the supernatant (S) were analyzed by Western blotting and the relative distribution of each protein in its respective fraction was determined. The relative distribution value was determined by dividing the band intensity of a protein in the fraction by the total (sum of the intensity value of that protein, both S and P fractions), and is expressed as a % of the total. Values with S.E. are an average of three determinations from three cultures. $^*p< 0.05$ with respect to the P fraction of the Ln-vector control. (B) Relative amount. The relative amounts of polymerized microtubules are relative distribution values from the microtubule pellet in panel (A) and are expressed as the % of Ln-vector control. Likewise, the relative amounts of microtubule-bound tau are the values of total tau in microtubule pellet in panel (A), and are expressed as a % of Ln-vector control. The relative amount of Ser^{262} phosphorylated tau was determined by normalizing the Ser^{262} blot by the corresponding tau blot, as in Figure 1. Values are average of three determinations from three cultures. $^*p<0.05$ with respect to the Ln-vector control.

doi: 10.1371/journal.pone.0084615.g004

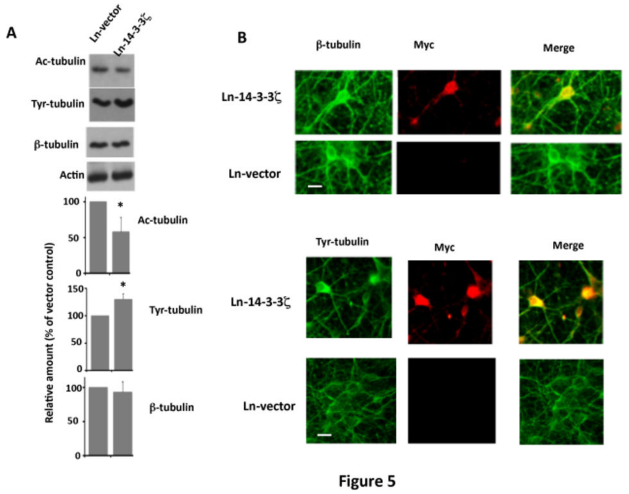

Figure 5. Overexpression of 14-3-3ζ in primary neurons in culture causes microtubule instability. Neurons infected with Ln-14-3-3ζ or Ln-vector were analyzed for microtubule instability by Western blotting or immunocytochemistry. (A) Western blot analysis. Western blot analysis for Ac-tubulin (stable microtubules), Tyr-tubulin (unstable microtubules) or β-tubulin (total tubulin) was performed. The Ac-tubulin or Tyr-tubulin band of each sample was normalized against the respective total tubulin band to determine the corresponding relative amount. To determine the relative amount of total tubulin, the tubulin band was normalized against the respective actin band. Values with standard error are the average of three determinations from three cultures. $^*p< 0.05$ with respect to Ln-vector infected controls. (B) Immunocytochemistry. Representative immunofluorescence micrographs of infected neurons immunostained with anti-β-tubulin (total tubulin), anti-Myc (Myc-14-3-3ζ), or anti-Tyr-tubulin. Scale bar. 25 μm.

doi: 10.1371/journal.pone.0084615.g005

culture develop synapses similar to those observed in the CNS, and neurons that are 3 weeks in culture have fully formed synapses. Synaptophysin protein level is widely used as a synaptic marker [71]. Therefore, we evaluated the effect of 14-3-3ζ overexpression on synapses by measuring synaptophysin protein level (Figure 6). Ln-vector infected neurons displayed numerous synaptophysin clusters in the cell soma and neurites, as expected (Figure 6A). In Ln-14-3-3ζ infected neurons, however, the number of labeled synaptophysin clusters was 52% less than in those expressing Ln-vector. In addition, in Ln-14-3-3ζ infected neurons, the synaptophysin clusters were less intense and relatively smaller in size when compared with those infected with Ln-vector.

Western blot analysis determined that, compared to Ln-vector infected neurons, the intensity of the synaptophysin protein band was significantly less in Ln-14-3-3ζ-infected neurons (compare lane 2 with lanes 3 and 4 in Figure 6B). Similar observations were made when another anti-synaptophysin antibody was used (Figure 6B, upper panel). 14-3-3ζ overexpression did not, however, affect the levels of post-synaptic marker protein, PSD-95, or the synaptic adhesion

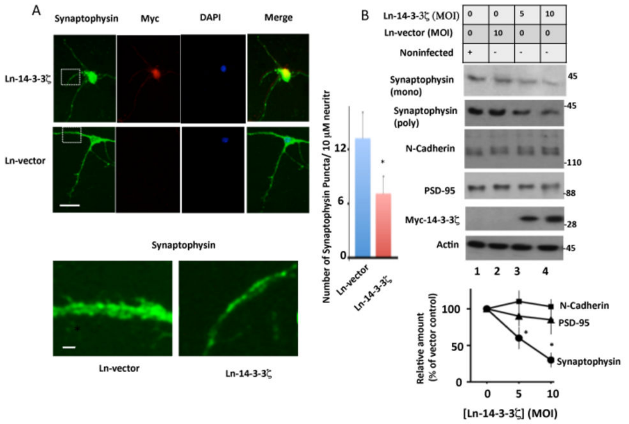

Figure 6

Figure 6. Overexpression of 14-3-3ζ downregulates synaptophysin protein level in rat hippocampal primary neurons in culture. – Neurons infected with Ln-14-3-3ζ or Ln-vector were analyzed for synaptophysin protein levels by immunocytochemistry or Western blotting. (A) Immunocytochemistry. Representative immunofluorescent micrographs are of neurons infected with the indicated virus and stained for Myc (14-3-3ζ), synaptophysin, DAPI (nucleus), and merge (co-localization). The corresponding inset is shown in higher magnification in the lower panel. Quantification of synaptophysin puncta from 50 neurites in each group from three different cultures is shown on the right hand side panel. Scale bars: upper 15 μm; lower, 5 μm. **p*<0.005 with respect to Ln-vector infected neurons. (B) Western blot analysis. Representative Western blots from extracts of neurons infected with Ln-14-3-3ζ or Ln-vector showing the level of indicated synaptic proteins in each culture. The relative amount of each protein was determined from the blots by normalizing the band intensity of that protein against the respective actin band. Values with standard error are an average of three determinations from three cultures. **p*< 0.05 with respect to Ln-vector infected cells.

doi: 10.1371/journal.pone.0084615.g006

protein N-cadherin, in neurons (Figure 6B). Based on this result, we concluded that 14-3-3ζ overexpression specifically downregulates synaptophysin protein level in rat hippocampal primary neurons in culture.

14-3-3ζ Overexpression Promotes Proteosomal Degradation of Synaptophysin in Neurons

To determine if 14-3-3ζ overexpression suppresses synaptophysin transcription, we isolated mRNA from Ln-vector and Ln-14-3-3ζ -infected neurons and performed quantitative real-time PCR as described in Materials and Methods. The level of synaptophysin mRNA in Ln-14-3-3ζ infected neurons was similar (88% ± 20) to that observed in Ln-vector-infected neurons (Figure S3). This result indicated that 14-3-3ζ overexpression does not affect synaptophysin gene expression in rat primary neurons.

In primary neurons, synaptophysin level is controlled both by mRNA translation and protein degradation [72,73]. To determine by which of the above mechanisms 14-3-3ζ down regulates synaptophysin level, we treated 14-3-3ζ-infected neurons with the protein synthesis inhibitor cycloheximide and monitored synaptophysin protein levels. As shown in Figure 7A, in Ln-vector infected neurons, synaptophysin level progressively decreased with an increase in post-drug treatment time (lanes 1-6) and became almost undetectable in 24 hr (lane 6). In 14-3-3ζ overexpressing neurons, synaptophysin level disappeared faster than in Ln-vector infected neurons (Figure 7A, lower). At the 2 hr time point, 28% of the total synaptophysin was degraded in 14-3-3ζ overexpressing neurons, whereas only 9% was degraded in the Ln-vector infected neurons. At the 4 and 8 hr time points, this value increased to 55 and 76%, respectively, in 14-3-3ζ expressing neurons, and 28 and 47%, respectively, in the vector expressing neurons. Thus, despite the blockage of protein synthesis, synaptophysin turnover continued to occur in both vector and 14-3-3ζ infected neurons. More importantly, in 14-3-3ζ expressing neurons, synaptophysin turnover was significantly faster than in vector expressing neurons (Figure 7A, lower). This result determined that 14-3-3ζ overexpression accelerated synaptophysin protein degradation in primary neurons in culture.

Lysosome and proteosome are the two major intracellular protein degrading systems in eukaryotic cells. To determine which of the two systems is involved in synaptophysin degradation, we treated Ln-14-3-3ζ infected neurons with lysosome inhibitor bafilomycin or NH_4Cl (Figure S4). The level of synaptophysin protein in 14-3-3ζ-infected and bafinomycin treated neurons was similar to those infected with Ln-14-3-3ζ and treated with vehicle (compare lane 2 with lane 4) or NH_4Cl (compare lane 2 with lane 6). This result excluded the involvement of lysosomes.

To evaluate if the proteosome is involved, we treated neurons overexpressing 14-3-3ζ with the proteosome inhibitor MG132 or lactacystin [53]. MG132 (carbobenzoxy-Leu-Leu-leucinal) is a peptide aldehyde that effectively blocks the proteolytic activity of the 26S proteosome complex. Lactacystin is structurally different from MG132 and is more specific, inhibiting proteosome function by acting as a pseudosubstrate and becoming covalently linked to the active site of the 20S subunit [74]. In neurons infected with Ln-14-3-3ζ and treated with MG132, the synaptophysin protein level was 2.6-fold more than in those infected with Ln-14-3-3ζ and treated with vehicle (Figure 7B, lane 3). Likewise, lactacystin treatment increased synaptophysin protein level in 14-3-3ζ expressing neurons by 2.9-fold (Figure 7B, lane 4). Based on these data, we concluded that overexpression of 14-3-3ζ promotes proteosomal degradation of synaptophysin in rat hippocampal primary neurons in culture.

14-3-3ζ Promotes Proteosomal Degradation of Synaptophysin by Destabilizing Microtubules in Neurons

Tau phosphorylation activates the proteosome in HEK-293 cells [75], and microtubule disruption leads to enhanced

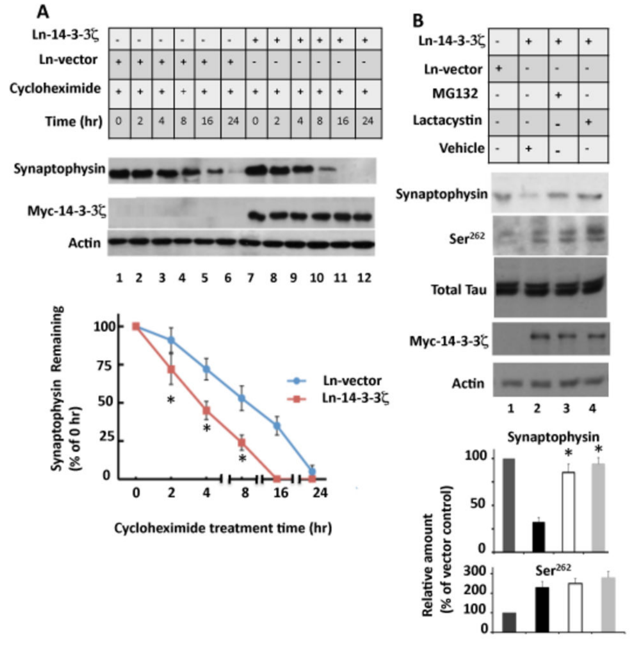

Figure 7

Figure 7. Overexpression of 14-3-3ζ in rat primary hippocampal neurons promotes proteosomal degradation of synaptophysin protein. (A) 14-3-3ζ overexpression does not affect synaptophysin protein synthesis in neurons. Ln-14-3-3ζ or Ln-vector infected neurons were treated with cycloheximide or vehicle for the indicated time and analyzed for synaptophysin protein by Western blotting. Synaptophysin band intensity in each lane was normalized against the corresponding actin band and is expressed as the % of 0 hr. Values with ± SE are the average of three determinations. **p*<0.005 with respect to Ln-vector infected neurons. (B) Proteosome inhibitors block the downregulation of synaptophysin protein caused by 14-3-3ζ overexpression. Primary neurons infected with Ln-14-3-3ζ or Ln-vector were treated with MG132 or lactacystin for 24 hr. Treated neurons were analyzed by Western blotting and the relative amounts were determined as per Figure 6. Values with standard error are the average of three determinations from three cultures. **p*< 0.005 with respect to Ln-14-3-3ζ infected and vehicle treated neurons.

doi: 10.1371/journal.pone.0084615.g007

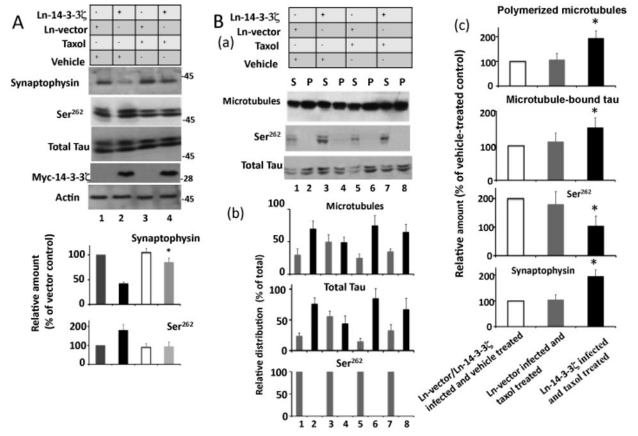

Figure 8

Figure 8. Microtubule stabilizing drug taxol restores synaptophysin protein levels in 14-3-3ζ overexpressing neurons. Neurons infected with Ln-14-3-3ζ or Ln-vector were treated with the microtubule stabilizing drug taxol for 24 hr and then analyzed by Western blotting to determine the relative amounts, and then subjected to a microtubule sedimentation assay. (A) Western blot analysis. The relative amount of each protein shown in the lower panel was determined as per Figure 5. Data with standard error are the average of three determinations from three cultures. **p* < 00.1 with respect to 14-3-3ζ infected and vehicle treated neurons. (B) Microtubule sedimentation assay. The microtubule sedimentation assay was performed as in Figure 4. The resulting microtubule pellet (P) and the supernatant (S) were analyzed by Western blotting and the relative distribution and relative amounts were determined as in Figure 4. (a) Western blots. (b), relative distribution. Values with S.E. are average of three determinations from three cultures. **p*< 0.05 with respect to the P fraction of Ln-vector control. (c) Relative amounts. The relative amount of polymerized microtubules are relative distribution values from the microtubule pellet in panel (b) and are expressed as the % of Ln-vector control. Likewise, the relative amounts of microtubule-bound tau are the values of total tau in the microtubule pellet in panel (b) and are expressed as a % of Ln-vector control. The relative amount of Ser^{262} phosphorylated tau was determined by normalizing Ser^{262} blot by corresponding tau blot as in Figure 4. Values are an average of three determinations from three cultures. **p*<0.05 with respect to Ln-vector control.

doi: 10.1371/journal.pone.0084615.g008

proteosomal activity in neurons [76]. 14-3-3ζ promotes tau phosphorylation at Ser^{262} and destabilizes microtubules (Figures 3-5). Therefore, it is possible that 14-3-3ζ promotes proteosomal degradation of synaptophysin by destabilizing microtubules. To test this possibility, we treated Ln-14-3-3ζ infected neurons with the microtubule stabilizing drug taxol, and then analyzed them.

Western blot analysis showed that synaptophysin levels were reduced in Ln-14-3-3ζ-infected neurons (Figure 8A, lane 2). Taxol treated neurons that were infected with Ln-14-3-3ζ increased synaptophysin protein levels 2.1-fold compared to vehicle-treated controls (Figure 8A, lane 4). In addition, taxol reduced tau phosphorylation at Ser^{262} by 2.1-fold in Ln-14-3-3ζ infected neurons (lane 4). Thus, taxol treatment reduced tau phosphorylation at Ser^{262} and provided significant protection against downregulation of synaptophysin induced by 14-3-3ζ overexpression.

To get more insight into the effects of taxol in Ln-14-3-3ζ overexpressing neurons, we performed a microtubule sedimentation assay. As shown in Figure 8B, compared to Ln-vector infected and vehicle-treated neurons, Ln-14-3-3ζ infected and vehicle-treated neurons had significantly lower

amounts of polymerized microtubules in the pellet (compare lane 4 with lane 2). Similarly, the amount of tau in the microtubule pellet was significantly less in Ln-14-3-3ζ infected and vehicle-treated neurons than in those infected with Ln-vector and treated with vehicle (compare lane 4 with lane 2). This observation is consistent with Figure 4 data and showed that 14-3-3ζ overexpression reduces the amount of microtubule-bound tau as well as the amount of polymerized microtubules in neurons.

More importantly, the amount of polymerized microtubules, microtubule-bound tau, Ser^{262} phosphorylated tau, and synaptophysin were similar in Ln-vector infected and vehicle-treated as well as Ln-vector infected and taxol treated neurons (Figure 8B (c)). However, compared to Ln-14-3-3ζ infected and vehicle treated neurons, Ln-14-3-3ζ infected and taxol treated neurons had almost 2-fold more polymerized microtubules, and microtubule-bound tau was 1.5 fold higher. This result indicates that taxol also protects neurons from 14-3-3ζ-induced loss of polymerized microtubules and reduction in the amount of microtubule bound tau.

Discussion

Tau is phosphorylated at multiple Ser/Thr sites which are divided into two classes: proline-directed and non proline-directed [35]. Proline-directed sites are phosphorylated by proline-directed kinases such as Cdk1, Cdk2, MAPK, Cdk5 and GSK3β. Non proline-directed sites are phosphorylated by PKA, PKC, CamKII, MARK, and phosphorylase kinase [25,62]. These kinases are activated by different stimuli and regulate different cellular functions. Although tau phosphorylation has mainly been implicated to regulate microtubule dynamics, current studies suggest that it is also involved in signal transduction [77].

In proliferating PC12 cells, tau is phosphorylated on all sites examined (Figure 1A, lane 1). When these cells are treated with NGF, phosphorylation at proline-directed sites $Ser^{396/404}$ and $Ser^{202/205}$ increases first (Figure 1). During this period, kinases are activated which then phosphorylate their respective cytosolic and nuclear substrates, and the NGF-induced signal propagates from the cell surface to the cytosol. Consequently, cells commit to differentiation [43]. With increasing time of NGF exposure, tau phosphorylation at proline-directed sites declines but is maintained at basal level, and non-proline-directed Ser^{262} phosphorylation becomes more and more prominent (Figure 1). During this period, neurites are formed and extended [78]. Neurite formation and extension requires the assembly of microtubules that run longitudinally within the neurite shaft [40,78]. Our data suggest that during neuronal differentiation, proline-directed tau phosphorylation is involved in signal propagation from the cell surface. Phosphorylation at Ser^{262}, on the other hand, regulates microtubule dynamics during neurite outgrowth and growth cone formation.

When NGF-exposed PC12 cells are treated with the PKA inhibitor P9115 or transfected with PKA-DN, Ser^{262} phosphorylation is significantly inhibited (Figures 1, 2). This data indicates that NGF promotes Ser^{262} phosphorylation by PKA. Surprisingly however, NGF does not activate PKA in these cells (Figure 2A). Previous studies have shown that PC12 cells have a basal level of PKA activity [63], and that when these cells are treated with NGF, expression of 14-3-3ζ is increased many fold, activating 14-3-3ζ-dependent events [66]. 14-3-3ζ is an adapter protein that regulates the function of its target proteins by binding to them [45,46]. *In vitro*, 14-3-3ζ binds to tau and promotes PKA-catalyzed tau Ser^{262} phosphorylation [62]. These observations and our data together suggest that NGF promotes Ser^{262} phosphorylation of tau by PKA in PC12 cells via enhancing intracellular 14-3-3ζ levels.

Abnormal tau phosphorylation is suggested to be fundamental to the development of NFT pathology in AD [1,2,17]. Although tau is phosphorylated at a number of sites, current studies indicate that phosphorylation at Ser^{262} has a higher pathological impact. In mouse brain and drosophila, both β-amyloid peptide and DNA damage cause neurodegeneration by promoting Ser^{262} phosphorylation [15,29]. In rat primary neurons in culture, accumulation of Ser^{262} phosphorylated tau causes synapse loss that is evident by the reduction of pre- and postsynaptic proteins [16]. The proteoglycan heparin, implicated to cause tau fibrillization in AD brain [79], promotes tau phosphorylation at Ser^{262} in vitro [54]. Neurotoxin MPTP promotes tau phosphorylation at Ser^{262} in human neuroblastoma cells [55]. Likewise α-synuclein, a component of senile plaques [80], promotes tau phosphorylation at Ser^{262} [55]. Sporadic AD is suggested to be multifactorial [1]. As discussed above, diverse factors that are implicated in causing AD pathology act via promotion of tau phosphorylation at Ser^{262}. These observations highlight the importance of Ser^{262} phosphorylation in AD pathogenesis.

The presence of 14-3-3ζ within the NFTs of AD brains has been reported by a number of studies [68,69]. Various stress-related genes in AD brain were examined, and out of 236 genes analyzed, the expression of 14-3-3ζ was found to be upregulated most significantly [49]. A profound increase in 14-3-3ζ expression was seen in areas affected by NFTs. This study showed that 14-3-3ζ upregulation is an early event and correlates with the severity of AD pathology. Recently, a number of studies have shown that 14-3-3ζ binds to tau and promotes tau phosphorylation and aggregation in vitro [62,81,82,83]. In this study, we showed that overexpression of 14-3-3ζ in rat primary neurons in culture promotes Ser^{262} phosphorylation. Together, these observations suggest that 14-3-3ζ may play a role in the development of AD pathology.

Synapses are the structural units of neuronal communication and play an essential role in learning and memory. Synapses are formed, matured, stabilized, remodeled, and eliminated throughout adulthood. Progressive loss of synapses is the major substrate of cognitive decline in patients suffering from AD [30,84]. Synapse loss begins early in the MCI stage and progresses along with the severity of the disease [50]. Biochemical, electron microscopy, and immunohistochemical studies have shown that the synaptic pathology in AD brain originates from the presynaptic terminal and spreads to postsynaptic sites. This is evident by the progressive decline in the level of presynaptic protein synaptophysin levels in the

brain [85]. Similar observations have been made in AD mouse models [86].

To determine the impact of increased expression of 14-3-3ζ in synapses, we analyzed three synaptic proteins in neurons infected with Ln-14-3-3ζ: presynaptic protein synaptophysin [87], postsynaptic protein PSD-95 [88], and intersynaptic adhesion protein N-Cadherin [89]. We found that 14-3-3ζ overexpression does not affect the levels of PSD-95 or the intracellular levels of N-Cadherin. However, neurons overexpressing 14-3-3ζ displayed significantly reduced synaptophysin levels due to accelerated degradation (Figure 7). A previous study has shown that 14-3-3ζ binds to the cell adhesion molecule L1 and negatively regulates neurite outgrowth in primary neurons in culture [48]. Synaptophysin is a synaptic vesicle protein regulating neurotransmitter release and synaptic plasticity, and is involved in synapse formation [87]. L1, on the other hand, is involved in axonal guidance [90]. Increased expression of 14-3-3ζ in AD brain may, therefore, cause synaptic pathology by inhibiting neurite outgrowth, synapse formation, and synaptic transmission.

Tau binds to microtubules and promotes microtubule formation *in vitro*. In this study, we found that, compared to Ln-vector infected neurons, the amount of microtubule bound tau is 30% less in Ln-14-3-3ζ infected neurons (Figure 4). This data indicates that overexpression of 14-3-3ζ in neurons inhibits tau binding to microtubules. Phosphorylation at Ser^{262} alone was shown to be sufficient to cause dissociation of tau from microtubules *in vitro* [21], and the level of Ser^{262} phosphorylated tau in 14-3-3ζ overexpressing neurons is 40% more than in those expressing Ln-vector (Figure 3). It is, therefore, likely that in neurons, 14-3-3ζ inhibits tau microtubule binding in part by promoting tau phosphorylation at Ser^{262}.

Overexpression of 14-3-3ζ in neurons causes phosphorylation of tau at Ser^{262}, destabilizes microtubules, and reduces intracellular synaptophysin levels (Figures 3, 4, 6 and 7). When 14-3-3ζ overexpressing neurons are treated with proteosome inhibitor, tau phosphorylation is not affected, but synaptophysin levels are restored (Figure 7). When 14-3-3ζ overexpressing neurons are treated with the microtubule stabilizing drug taxol, both tau Ser^{262} phosphorylation and synaptophysin degradation are inhibited (Figure 8). Phosphorylation at Ser^{262} destabilizes microtubules [21], and microtubule instability activates the proteosome [76]. It is, therefore, possible that 14-3-3ζ destabilizes microtubules by promoting tau phosphorylation at Ser^{262}, and microtubule destabilization then accelerates proteosomal degradation of synaptophysin.

A recent study has reported that transgenic overexpression of 14-3-3ζ protects the hippocampus against endoplasmic reticulum stress and epileptic seizures in mouse brain [91]. In this study, the authors show that overexpression of 14-3-3ζ in the brain prevents neuronal apoptosis caused by seizure. We have found that when overexpressed in primary neurons in culture, 14-3-3ζ does not promote apoptosis (Figure 2). However, 14-3-3ζ overexpression significantly downregulates synaptophysin protein levels (Figure 6), suggesting its involvement in synapse elimination. It is possible that 14-3-3ζ may exert its anti-apoptotic effects and downregulation of synaptophysin protein activities via independent mechanisms.

Increased expression of various 14-3-3 isoforms is observed in a number of human diseases: 14-3-3σ in colorectal carcinoma [92], 14-3-3β, γ, ζ and τ in lung cancers [93,94], 14-3-3τ in breast cancer [95], 14-3-3β, γ, η (τ), σ and ε in the reactive astrocytes of Creutzfeldt-Jakob disease brain [96], 14-3-3γ in ischemic brain [97,98], 14-3-3τ in amyotrophic lateral sclerosis spinal cord [99], 14-3-3ε in reactive astrocytes of demyelinating lesions of Multiple Sclerosis [100], and 14-3-3ζ in AD brain [49]. The pathological significance of the increased 14-3-3 isoforms in these various disorders is not entirely clear. However, 14-3-3 proteins have been suggested to be oncogenic [101]. Overexpression of 14-3-3τ in breast carcinoma cells promotes proteosomal degradation of Cdk inhibitor p21, which is one of the major regulators of p53-dependent tumor suppression and cellular senescence [95]. In this study, we showed that overexpression of 14-3-3ζ in primary neurons in culture promotes proteosomal degradation of presynaptic protein synaptophysin. It will be interesting to examine if activation of the proteosome is a common mechanism in various human diseases associated with increased levels of different 14-3-3 isoforms.

Supporting Information

Figure S1. Disruption of PKA activity inhibits NGF-induced tau Ser^{262} phosphorylation in PC12 cells. PC12 cells transfected with Myc-PKA-DN or vector were exposed to NGF and then analyzed for tau phosphorylation by Western blot analysis. Based on blot band intensities, relative amount of tau phosphorylation at indicated sites was determined. Values are mean ± S.E. from three determinations. *$p< 0.005$ with respect to vector transfected and NGF exposed cells.
(TIF)

Figure S2. Overexpression of 14-3-3ζ does not affect survival of neurons. Rat hippocamal neurons in culture were infected with Ln-14-3-3ζ or Ln-vector and then analyzed by MTT assay for cell survival (panel A) or by Western blot analysis for active caspase 3. Neurons treated with staurosporine (2 mg/ml) were used as positive controls.
(TIF)

Figure S3. 14-3-3ζ overexpression does not affect synaptophysin transcription in neurons. Ln-14-3-3ζ or Ln-vector infected rat hippocampal primary neurons in culture were analyzed for the level of synaptophysin mRNA by qRT-PCR.
(TIF)

Figure S4. Synaptophysin degrdation in 14-3-3ζ overexpressing neurons in not mediated by lysosome. Ln-vector or Ln-14-3-3ζ infected neurons were treated with indicated lysome inhibitor and analyzed by Western blot analysis.
(TIF)

Acknowledgements

Ryen MacDonald is recipient of a PhD fellowships from Alzheimer's Society of Canada.

Author Contributions

Conceived and designed the experiments: HQ DH HP. Performed the experiments: HQ DH. Analyzed the data: HQ DH HP RM. Contributed reagents/materials/analysis tools: HP. Wrote the manuscript: HQ DH HP RM.

References

1. Lee VM, Goedert M, Trojanowski JQ (2001) Neurodegenerative tauopathies. Annu Rev Neurosci 24: 1121-1159. doi:10.1146/annurev.neuro.24.1.1121. PubMed: 11520930.
2. Lee G, Leugers CJ (2012) Tau and tauopathies. Prog Mol Biol. Transl Sci 107: 263-293.
3. Hong M, Zhukareva V, Vogelsberg-Ragaglia V, Wszolek Z, Reed L et al. (1998) Mutation-specific functional impairments in distinct tau isoforms of hereditary FTDP-17. Science 282: 1914-1917. doi:10.1126/science.282.5395.1914. PubMed: 9836646.
4. Vogelsberg-Ragaglia V, Bruce J, Richter-Landsberg C, Zhang B, Hong M et al. (2000) Distinct FTDP-17 missense mutations in tau produce tau aggregates and other pathological phenotypes in transfected CHO cells. Mol Biol Cell 11: 4093-4104. doi:10.1091/mbc.11.12.4093. PubMed: 11102510.
5. Lim F, Hernández F, Lucas JJ, Gómez-Ramos P, Morán MA et al. (2001) FTDP-17 mutations in tau transgenic mice provoke lysosomal abnormalities and Tau filaments in forebrain. Mol Cell Neurosci 18: 702-714. doi:10.1006/mcne.2001.1051. PubMed: 11749044.
6. von Bergen M, Barghorn S, Li L, Marx A, Biernat J et al. (2001) Mutations of tau protein in frontotemporal dementia promote aggregation of paired helical filaments by enhancing local beta-structure. J Biol Chem 276: 48165-48174. PubMed: 11606569.
7. Allen B, Ingram E, Takao M, Smith MJ, Jakes R et al. (2002) Abundant tau filaments and nonapoptotic neurodegeneration in transgenic mice expressing human P301S tau protein. J Neurosci 22: 9340-9351. PubMed: 12417659.
8. Tanemura K, Murayama M, Akagi T, Hashikawa T, Tominaga T et al. (2002) Neurodegeneration with tau accumulation in a transgenic mouse expressing V337M human tau. J Neurosci 22: 133-141. PubMed: 11756496.
9. Alonso Adel C, Mederlyova A, Novak M, Grundke-Iqbal I, Iqbal K (2004) Promotion of hyperphosphorylation by frontotemporal dementia tau mutations. J Biol Chem 279: 34873-34881. doi:10.1074/jbc.M405131200. PubMed: 15190058.
10. Santacruz K, Lewis J, Spires T, Paulson J, Kotilinek L et al. (2005) Tau suppression in a neurodegenerative mouse model improves memory function. Science 309: 476-481. doi:10.1126/science.1113694. PubMed: 16020737.
11. Han D, Qureshi HY, Lu Y, Paudel HK (2009) Familial FTDP-17 missense mutations inhibit microtubule assembly-promoting activity of tau by increasing phosphorylation at Ser202 in vitro. J Biol Chem 284: 13422-13433. doi:10.1074/jbc.M901095200. PubMed: 19304664.
12. Brunden KR, Trojanowski JQ, Lee VM (2009) Advances in tau-focused drug discovery for Alzheimer's disease and related tauopathies. Nat Rev Drug Discov 8: 783-793. doi:10.1038/nrd2959. PubMed: 19794442.
13. Rapoport M, Dawson HN, Binder LI, Vitek MP, Ferreira A (2002) Tau is essential to beta -amyloid-induced neurotoxicity. Proc Natl Acad Sci U S A 99: 6364-6369. doi:10.1073/pnas.092136199. PubMed: 11959919.
14. Roberson ED, Scearce-Levie K, Palop JJ, Yan F, Cheng IH et al. (2007) Reducing endogenous tau ameliorates amyloid beta-induced deficits in an Alzheimer's disease mouse model. Science 316: 750-754. doi:10.1126/science.1141736. PubMed: 17478722.
15. Iijima K, Gatt A, Iijima-Ando K (2010) Tau Ser262 phosphorylation is critical for Abeta42-induced tau toxicity in a transgenic Drosophila model of Alzheimer's disease. Hum Mol Genet 19: 2947-2957. doi:10.1093/hmg/ddq200. PubMed: 20466736.
16. Yu W, Polepalli J, Wagh D, Rajadas J, Malenka R et al. (2012) A critical role for the PAR-1/MARK-tau axis in mediating the toxic effects of Abeta on synapses and dendritic spines. Hum Mol Genet 21: 1384-1390. doi:10.1093/hmg/ddr576. PubMed: 22156579.
17. Alonso AC, Zaidi T, Grundke-Iqbal I, Iqbal K (1994) Role of abnormally phosphorylated tau in the breakdown of microtubules in Alzheimer disease. Proc Natl Acad Sci U S A 91: 5562-5566. doi:10.1073/pnas.91.12.5562. PubMed: 8202528.
18. Morishima-Kawashima M, Hasegawa M, Takio K, Suzuki M, Yoshida H et al. (1995) Proline-directed and non-proline-directed phosphorylation of PHF-tau. J Biol Chem 270: 823-829. doi:10.1074/jbc.270.2.823. PubMed: 7822317.
19. Seubert P, Mawal-Dewan M, Barbour R, Jakes R, Goedert M et al. (1995) Detection of phosphorylated Ser262 in fetal tau, adult tau, and paired helical filament tau. J Biol Chem 270: 18917-18922. doi:10.1074/jbc.270.32.18917. PubMed: 7642549.
20. Hanger DP, Byers HL, Wray S, Leung KY, Saxton MJ et al. (2007) Novel phosphorylation sites in tau from Alzheimer brain support a role for casein kinase 1 in disease pathogenesis. J Biol Chem 282: 23645-23654. doi:10.1074/jbc.M703269200. PubMed: 17562708.
21. Biernat J, Gustke N, Drewes G, Mandelkow EM, Mandelkow E (1993) Phosphorylation of Ser262 strongly reduces binding of tau to microtubules: distinction between PHF-like immunoreactivity and microtubule binding. Neuron 11: 153-163. doi:10.1016/0896-6273(93)90279-Z. PubMed: 8393323.
22. Ahlijanian MK, Barrezueta NX, Williams RD, Jakowski A, Kowsz KP et al. (2000) Hyperphosphorylated tau and neurofilament and cytoskeletal disruptions in mice overexpressing human p25, an activator of cdk5. Proc Natl Acad Sci U S A 97: 2910-2915. doi:10.1073/pnas.040577797. PubMed: 10706614.
23. Cruz JC, Tseng HC, Goldman JA, Shih H, Tsai LH (2003) Aberrant Cdk5 activation by p25 triggers pathological events leading to neurodegeneration and neurofibrillary tangles. Neuron 40: 471-483. doi:10.1016/S0896-6273(03)00627-5. PubMed: 14642273.
24. Sakaue F, Saito T, Sato Y, Asada A, Ishiguro K et al. (2005) Phosphorylation of FTDP-17 mutant tau by cyclin-dependent kinase 5 complexed with p35, p25, or p39. J Biol Chem 280: 31522-31529. doi:10.1074/jbc.M504792200. PubMed: 15994305.
25. Hanger DP, Anderton BH, Noble W (2009) Tau phosphorylation: the therapeutic challenge for neurodegenerative disease. Trends Mol Med 15: 112-119. doi:10.1016/j.molmed.2009.01.003. PubMed: 19246243.
26. Lucas JJ, Hernández F, Gómez-Ramos P, Morán MA, Hen R et al. (2001) Decreased nuclear beta-catenin, tau hyperphosphorylation and neurodegeneration in GSK-3beta conditional transgenic mice. EMBO J 20: 27-39. doi:10.1093/emboj/20.1.27. PubMed: 11226152.
27. Sun W, Qureshi HY, Cafferty PW, Sobue K, Agarwal-Mawal A et al. (2002) Glycogen synthase kinase-3beta is complexed with tau protein in brain microtubules. J Biol Chem 277: 11933-11940. doi:10.1074/jbc.M107182200. PubMed: 11812770.
28. Sobue K, Agarwal-Mawal A, Li W, Sun W, Miura Y et al. (2000) Interaction of neuronal Cdc2-like protein kinase with microtubule-associated protein tau. J Biol Chem 275: 16673-16680. doi:10.1074/jbc.M000784200. PubMed: 10749861.
29. Nishimura I, Yang Y, Lu B (2004) PAR-1 kinase plays an initiator role in a temporally ordered phosphorylation process that confers tau toxicity in Drosophila. Cell 116: 671-682. doi:10.1016/S0092-8674(04)00170-9. PubMed: 15006350.
30. Selkoe DJ (2002) Alzheimer's disease is a synaptic failure. Science 298: 789-791. doi:10.1126/science.1074069. PubMed: 12399581.
31. Sironi JJ, Yen SH, Gondal JA, Wu Q, Grundke-Iqbal I et al. (1998) Ser-262 in human recombinant tau protein is a markedly more favorable site for phosphorylation by CaMKII than PKA or PhK. FEBS Lett 436: 471-475. doi:10.1016/S0014-5793(98)01185-5. PubMed: 9801171.
32. Drewes G, Ebneth A, Preuss U, Mandelkow EM, Mandelkow E (1997) MARK, a novel family of protein kinases that phosphorylate microtubule-associated proteins and trigger microtubule disruption. Cell 89: 297-308. doi:10.1016/S0092-8674(00)80208-1. PubMed: 9108484.
33. Litersky JM, Johnson GV, Jakes R, Goedert M, Lee M et al. (1996) Tau protein is phosphorylated by cyclic AMP-dependent protein kinase and calcium/calmodulin-dependent protein kinase II within its microtubule-binding domains at Ser-262 and Ser-356. Biochem J 316 (2): 655-660. PubMed: 8687413.
34. Paudel HK (1997) The regulatory Ser262 of microtubule-associated protein tau is phosphorylated by phosphorylase kinase. J Biol Chem 272: 1777-1785. PubMed: 8999860.

35. Watanabe A, Hasegawa M, Suzuki M, Takio K, Morishima-Kawashima M et al. (1993) In vivo phosphorylation sites in fetal and adult rat tau. J Biol Chem 268: 25712-25717. PubMed: 8245007.
36. Vincent I, Rosado M, Davies P (1996) Mitotic mechanisms in Alzheimer's disease? J Cell Biol 132: 413-425. doi:10.1083/jcb.132.3.413. PubMed: 8636218.
37. Preuss U, Mandelkow EM (1998) Mitotic phosphorylation of tau protein in neuronal cell lines resembles phosphorylation in Alzheimer's disease. Eur J Cell Biol 76: 176-184. doi:10.1016/S0171-9335(98)80032-0. PubMed: 9716264.
38. Illenberger S, Zheng-Fischhöfer Q, Preuss U, Stamer K, Baumann K et al. (1998) The endogenous and cell cycle-dependent phosphorylation of tau protein in living cells: implications for Alzheimer's disease. Mol Biol Cell 9: 1495-1512. doi:10.1091/mbc.9.6.1495. PubMed: 9614189.
39. Delobel P, Flament S, Hamdane M, Mailliot C, Sambo AV et al. (2002) Abnormal Tau phosphorylation of the Alzheimer-type also occurs during mitosis. J Neurochem 83: 412-420. doi:10.1046/j.1471-4159.2002.01143.x. PubMed: 12423251.
40. Schipper HM, Agarwal-Mawal A, Paudel HK (1999) The topography and subcellular distribution of mitogen-activated protein kinase kinase1 (MEK1) in adult rat brain and differentiating PC12 cells. Neuroscience 93: 585-595. doi:10.1016/S0306-4522(99)00120-7. PubMed: 10465442.
41. Li T, Chalifour LE, Paudel HK (2007) Phosphorylation of protein phosphatase 1 by cyclin-dependent protein kinase 5 during nerve growth factor-induced PC12 cell differentiation. J Biol Chem 282: 6619-6628. PubMed: 17202132.
42. Yan GZ, Ziff EB (1995) NGF regulates the PC12 cell cycle machinery through specific inhibition of the Cdk kinases and induction of cyclin D1. J Neurosci 15: 6200-6212. PubMed: 7666202.
43. Cowley S, Paterson H, Kemp P, Marshall CJ (1994) Activation of MAP kinase kinase is necessary and sufficient for PC12 differentiation and for transformation of NIH 3T3 cells. Cell 77: 841-852. doi:10.1016/0092-8674(94)90133-3. PubMed: 7911739.
44. Harada T, Morooka T, Ogawa S, Nishida E (2001) ERK induces p35, a neuron-specific activator of Cdk5, through induction of Egr1. Nat Cell Biol 3: 453-459. doi:10.1038/35074516. PubMed: 11331872.
45. Fu H, Subramanian RR, Masters SC Fu H, Subramanian RR, Masters SC (2000) 14-3-3 proteins: structure, function, and regulation. Annu Rev Pharmacol Toxicol 40: 617-647 doi:10.1146/annurev.pharmtox.40.1.617. PubMed: 10836149. doi:10.1146/annurev.pharmtox.40.1.617. PubMed: 10836149.
46. Berg D, Holzmann C, Riess O (2003) 14-3-3 proteins in the nervous system. Nat Rev Neurosci 4: 752-762. doi:10.1038/nrm1233. PubMed: 12951567.
47. Cheah PS, Ramshaw HS, Thomas PQ, Toyo-Oka K, Xu X et al. (2012) Neurodevelopmental and neuropsychiatric behaviour defects arise from 14-3-3zeta deficiency. Mol Psychiatry 17: 451-466. doi:10.1038/mp.2011.158. PubMed: 22124272.
48. Ramser EM, Wolters G, Dityateva G, Dityatev A, Schachner M et al. (2010) The 14-3-3zeta protein binds to the cell adhesion molecule L1, promotes L1 phosphorylation by CKII and influences L1-dependent neurite outgrowth. PLOS ONE 5: e13462. doi:10.1371/journal.pone.0013462. PubMed: 20976158.
49. Soulié C, Nicole A, Delacourte A, Ceballos-Picot I (2004) Examination of stress-related genes in human temporal versus occipital cortex in the course of neurodegeneration: involvement of 14-3-3 zeta in this dynamic process. Neurosci Lett 365: 1-5. doi:10.1016/j.neulet.2004.03.090. PubMed: 15234461.
50. Masliah E, Mallory M, Alford M, DeTeresa R, Hansen LA et al. (2001) Altered expression of synaptic proteins occurs early during progression of Alzheimer's disease. Neurology 56: 127-129. doi:10.1212/WNL.56.1.127. PubMed: 11148253.
51. Lu Y, Li T, Qureshi HY, Han D, Paudel HK (2011) Early growth response 1 (Egr-1) regulates phosphorylation of microtubule-associated protein tau in mammalian brain. J Biol Chem 286: 20569-20581. doi:10.1074/jbc.M111.220962. PubMed: 21489990.
52. Li T, Hawkes C, Qureshi HY, Kar S, Paudel HK (2006) Cyclin-dependent protein kinase 5 primes microtubule-associated protein tau site-specifically for glycogen synthase kinase 3beta. Biochemistry 45: 3134-3145. doi:10.1021/bi051635j. PubMed: 16519508.
53. Song W, Patel A, Qureshi HY, Han D, Schipper HM et al. (2009) The Parkinson disease-associated A30P mutation stabilizes alpha-synuclein against proteasomal degradation triggered by heme oxygenase-1 over-expression in human neuroblastoma cells. J Neurochem 110: 719-733. doi:10.1111/j.1471-4159.2009.06165.x. PubMed: 19457084.
54. Paudel HK, Li W (1999) Heparin-induced conformational change in microtubule-associated protein Tau as detected by chemical cross-linking and phosphopeptide mapping. J Biol Chem 274: 8029-8038. doi:10.1074/jbc.274.12.8029. PubMed: 10075702.
55. Qureshi HY, Paudel HK (2011) Parkinsonian neurotoxin 1-methyl-4-phenyl-1,2,3,6-tetrahydropyridine (MPTP) and alpha-synuclein mutations promote Tau protein phosphorylation at Ser262 and destabilize microtubule cytoskeleton in vitro. J Biol Chem 286: 5055-5068. doi:10.1074/jbc.M110.178905. PubMed: 21127069.
56. Li T, Paudel HK (2006) Glycogen synthase kinase 3beta phosphorylates Alzheimer's disease-specific Ser396 of microtubule-associated protein tau by a sequential mechanism. Biochemistry 45: 3125-3133. doi:10.1021/bi051634r. PubMed: 16519507.
57. Pap M, Cooper GM (1998) Role of glycogen synthase kinase-3 in the phosphatidylinositol 3-Kinase/Akt cell survival pathway. J Biol Chem 273: 19929-19932. doi:10.1074/jbc.273.32.19929. PubMed: 9685326.
58. Jicha GA, Weaver C, Lane E, Vianna C, Kress Y et al. (1999) cAMP-dependent protein kinase phosphorylations on tau in Alzheimer's disease. J Neurosci 19: 7486-7494. PubMed: 10460255.
59. Scott CW, Spreen RC, Herman JL, Chow FP, Davison MD et al. (1993) Phosphorylation of recombinant tau by cAMP-dependent protein kinase. Identification of phosphorylation sites and effect on microtubule assembly. J Biol Chem 268: 1166-1173. PubMed: 8419321.
60. Liu SJ, Zhang JY, Li HL, Fang ZY, Wang Q et al. (2004) Tau becomes a more favorable substrate for GSK-3 when it is prephosphorylated by PKA in rat brain. J Biol Chem 279: 50078-50088. doi:10.1074/jbc.M406109200. PubMed: 15375165.
61. Taylor SS, Buechler JA, Yonemoto W (1990) cAMP-dependent protein kinase: framework for a diverse family of regulatory enzymes. Annu Rev Biochem 59: 971-1005. doi:10.1146/annurev.bi.59.070190.004543. PubMed: 2165385.
62. Hashiguchi M, Sobue K, Paudel HK (2000) 14-3-3zeta is an effector of tau protein phosphorylation. J Biol Chem 275: 25247-25254. doi:10.1074/jbc.M003738200. PubMed: 10840038.
63. Cassano S, Gallo A, Buccigrossi V, Porcellini A, Cerillo R et al. (1996) Membrane localization of cAMP-dependent protein kinase amplifies cAMP signaling to the nucleus in PC12 cells. J Biol Chem 271: 29870-29875. doi:10.1074/jbc.271.47.29870. PubMed: 8939928.
64. Amieux PS, Howe DG, Knickerbocker H, Lee DC, Su T et al. (2002) Increased basal cAMP-dependent protein kinase activity inhibits the formation of mesoderm-derived structures in the developing mouse embryo. J Biol Chem 277: 27294-27304. doi:10.1074/jbc.M200302200. PubMed: 12004056.
65. Vinogradova TM, Lyashkov AE, Zhu W, Ruknudin AM, Sirenko S et al. (2006) High basal protein kinase A-dependent phosphorylation drives rhythmic internal Ca2+ store oscillations and spontaneous beating of cardiac pacemaker cells. Circ Res 98: 505-514. doi:10.1161/01.RES.0000204575.94040.d1. PubMed: 16424365.
66. Angelastro JM, Klimaschewski L, Tang S, Vitolo OV, Weissman TA et al. (2000) Identification of diverse nerve growth factor-regulated genes by serial analysis of gene expression (SAGE) profiling. Proc Natl Acad Sci U S A 97: 10424-10429. doi:10.1073/pnas.97.19.10424. PubMed: 10984536.
67. Hanger DP, Betts JC, Loviny TL, Blackstock WP, Anderton BH (1998) New phosphorylation sites identified in hyperphosphorylated tau (paired helical filament-tau) from Alzheimer's disease brain using nanoelectrospray mass spectrometry. J Neurochem 71: 2465-2476. PubMed: 9832145.
68. Layfield R, Fergusson J, Aitken A, Lowe J, Landon M et al. (1996) Neurofibrillary tangles of Alzheimer's disease brains contain 14-3-3 proteins. Neurosci Lett 209: 57-60. doi:10.1016/0304-3940(96)12598-2. PubMed: 8734909.
69. Umahara T, Uchihara T, Tsuchiya K, Nakamura A, Iwamoto T et al. (2004) 14-3-3 proteins and zeta isoform containing neurofibrillary tangles in patients with Alzheimer's disease. Acta Neuropathol 108: 279-286. doi:10.1007/s00401-004-0885-4. PubMed: 15235803.
70. Arendt T (2009) Synaptic degeneration in Alzheimer's disease. Acta Neuropathol 118: 167-179. doi:10.1007/s00401-009-0536-x. PubMed: 19390859.
71. Honer WG (2003) Pathology of presynaptic proteins in Alzheimer's disease: more than simple loss of terminals. Neurobiol Aging 24: 1047-1062. doi:10.1016/j.neurobiolaging.2003.04.005. PubMed: 14643376.
72. Daly C, Ziff EB (1997) Post-transcriptional regulation of synaptic vesicle protein expression and the developmental control of synaptic vesicle formation. J Neurosci 17: 2365-2375. PubMed: 9065497.
73. Wheeler TC, Chin LS, Li Y, Roudabush FL, Li L (2002) Regulation of synaptophysin degradation by mammalian homologues of seven in absentia. J Biol Chem 277: 10273-10282. doi:10.1074/jbc.M107857200. PubMed: 11786535.

74. Lee DH, Goldberg AL (1998) Proteasome inhibitors: valuable new tools for cell biologists. Trends Cell Biol 8: 397-403. doi:10.1016/S0962-8924(98)01346-4. PubMed: 9789328.
75. Ren QG, Liao XM, Chen XQ, Liu GP, Wang JZ (2007) Effects of tau phosphorylation on proteasome activity. FEBS Lett 581: 1521-1528. doi:10.1016/j.febslet.2007.02.065. PubMed: 17376439.
76. Huff LM, Sackett DL, Poruchynsky MS, Fojo T (2010) Microtubule-disrupting chemotherapeutics result in enhanced proteasome-mediated degradation and disappearance of tubulin in neural cells. Cancer Res 70: 5870-5879. doi:10.1158/0008-5472.CAN-09-4281. PubMed: 20587529.
77. Leugers CJ, Lee G (2010) Tau potentiates nerve growth factor-induced mitogen-activated protein kinase signaling and neurite initiation without a requirement for microtubule binding. J Biol Chem 285: 19125-19134. doi:10.1074/jbc.M110.105387. PubMed: 20375017.
78. Black MM, Aletta JM, Greene LA (1986) Regulation of microtubule composition and stability during nerve growth factor-promoted neurite outgrowth. J Cell Biol 103: 545-557. doi:10.1083/jcb.103.2.545. PubMed: 3733878.
79. Goedert M, Jakes R, Spillantini MG, Hasegawa M, Smith MJ et al. (1996) Assembly of microtubule-associated protein tau into Alzheimer-like filaments induced by sulphated glycosaminoglycans. Nature 383: 550-553. doi:10.1038/383550a0. PubMed: 8849730.
80. Iwai A (2000) Properties of NACP/alpha-synuclein and its role in Alzheimer's disease. Biochim Biophys Acta 1502: 95-109. doi:10.1016/S0925-4439(00)00036-3. PubMed: 10899435.
81. Sadik G, Tanaka T, Kato K, Yamamori H, Nessa BN et al. (2009) Phosphorylation of tau at Ser214 mediates its interaction with 14-3-3 protein: implications for the mechanism of tau aggregation. J Neurochem 108: 33-43. doi:10.1111/j.1471-4159.2008.05716.x. PubMed: 19014373.
82. Hernández F, Cuadros R, Avila J (2004) Zeta 14-3-3 protein favours the formation of human tau fibrillar polymers. Neurosci Lett 357: 143-146. doi:10.1016/j.neulet.2003.12.049. PubMed: 15036595.
83. Sluchanko NN, Sudnitsyna MV, Seit-Nebi AS, Antson AA, Gusev NB (2011) Properties of the monomeric form of human 14-3-3zeta protein and its interaction with tau and HspB6. Biochemistry 50: 9797-9808. doi:10.1021/bi201374s. PubMed: 21978388.
84. Terry RD, Masliah E, Salmon DP, Butters N, DeTeresa R et al. (1991) Physical basis of cognitive alterations in Alzheimer's disease: synapse loss is the major correlate of cognitive impairment. Ann Neurol 30: 572-580. doi:10.1002/ana.410300410. PubMed: 1789684.
85. Sze CI, Troncoso JC, Kawas C, Mouton P, Price DL et al. (1997) Loss of the presynaptic vesicle protein synaptophysin in hippocampus correlates with cognitive decline in Alzheimer disease. J Neuropathol Exp Neurol 56: 933-944. doi:10.1097/00005072-199708000-00011. PubMed: 9258263.
86. Hsia AY, Masliah E, McConlogue L, Yu GQ, Tatsuno G et al. (1999) Plaque-independent disruption of neural circuits in Alzheimer's disease mouse models. Proc Natl Acad Sci U S A 96: 3228-3233. doi:10.1073/pnas.96.6.3228. PubMed: 10077666.
87. Tarsa L, Goda Y (2002) Synaptophysin regulates activity-dependent synapse formation in cultured hippocampal neurons. Proc Natl Acad Sci U S A 99: 1012-1016. doi:10.1073/pnas.022575999. PubMed: 11792847.
88. El-Husseini AE, Schnell E, Chetkovich DM, Nicoll RA, Bredt DS (2000) PSD-95 involvement in maturation of excitatory synapses. Science 290: 1364-1368. PubMed: 11082065.
89. Mendez P, De Roo M, Poglia L, Klauser P, Muller D (2010) N-cadherin mediates plasticity-induced long-term spine stabilization. J Cell Biol 189: 589-600. doi:10.1083/jcb.201003007. PubMed: 20440002.
90. Schmid RS, Maness PF (2008) L1 and NCAM adhesion molecules as signaling coreceptors in neuronal migration and process outgrowth. Curr Opin Neurobiol 18: 245-250. doi:10.1016/j.conb.2008.07.015. PubMed: 18760361.
91. Brennan GP, Jimenez-Mateos EM, McKiernan RC, Engel T, Tzivion G et al. (2013) Transgenic overexpression of 14-3-3 zeta protects hippocampus against endoplasmic reticulum stress and status epilepticus in vivo. PLOS ONE 8: e54491. doi:10.1371/journal.pone.0054491. PubMed: 23359526.
92. Perathoner A, Pirkebner D, Brandacher G, Spizzo G, Stadlmann S et al. (2005) 14-3-3sigma expression is an independent prognostic parameter for poor survival in colorectal carcinoma patients. Clin Cancer Res 11: 3274-3279. doi:10.1158/1078-0432.CCR-04-2207. PubMed: 15867223.
93. Qi W, Liu X, Qiao D, Martinez JD (2005) Isoform-specific expression of 14-3-3 proteins in human lung cancer tissues. Int J Cancer 113: 359-363. doi:10.1002/ijc.20492. PubMed: 15455356.
94. Fan T, Li R, Todd NW, Qiu Q, Fang HB et al. (2007) Up-regulation of 14-3-3zeta in lung cancer and its implication as prognostic and therapeutic target. Cancer Res 67: 7901-7906. doi:10.1158/0008-5472.CAN-07-0090. PubMed: 17699796.
95. Wang B, Liu K, Lin HY, Bellam N, Ling S et al. (2010) 14-3-3Tau regulates ubiquitin-independent proteasomal degradation of p21, a novel mechanism of p21 downregulation in breast cancer. Mol Cell Biol 30: 1508-1527. doi:10.1128/MCB.01335-09. PubMed: 20086099.
96. Kawamoto Y, Akiguchi I, Jarius C, Budka H (2004) Enhanced expression of 14-3-3 proteins in reactive astrocytes in Creutzfeldt-Jakob disease brains. Acta Neuropathol 108: 302-308. doi:10.1007/s00401-004-0892-5. PubMed: 15235801.
97. Chen XQ, Chen JG, Zhang Y, Hsiao WW, Yu AC (2003) 14-3-3gamma is upregulated by in vitro ischemia and binds to protein kinase Raf in primary cultures of astrocytes. Glia 42: 315-324. doi:10.1002/glia.10185. PubMed: 12730952.
98. Pirim I (1998) Ischemic rat brains contain immunoreactivity of 14-3-3 proteins. Int J Neurosci 95: 101-106. doi:10.3109/00207459809000653. PubMed: 9845020.
99. Malaspina A, Kaushik N, de Belleroche J (2000) A 14-3-3 mRNA is up-regulated in amyotrophic lateral sclerosis spinal cord. J Neurochem 75: 2511-2520. PubMed: 11080204.
100. Satoh J, Yamamura T, Arima K (2004) The 14-3-3 protein epsilon isoform expressed in reactive astrocytes in demyelinating lesions of multiple sclerosis binds to vimentin and glial fibrillary acidic protein in cultured human astrocytes. Am J Pathol 165: 577-592. doi:10.1016/S0002-9440(10)63322-6. PubMed: 15277231.
101. Tzivion G, Gupta VS, Kaplun L, Balan V (2006) 14-3-3 proteins as potential oncogenes. Semin Cancer Biol 16: 203-213. doi:10.1016/j.semcancer.2006.03.004. PubMed: 16725345.
